# On the Structure and Function of the Phytoene Desaturase CRTI from *Pantoea ananatis*, a Membrane-Peripheral and FAD-Dependent Oxidase/Isomerase

**DOI:** 10.1371/journal.pone.0039550

**Published:** 2012-06-22

**Authors:** Patrick Schaub, Qiuju Yu, Sandra Gemmecker, Pierre Poussin-Courmontagne, Justine Mailliot, Alastair G. McEwen, Sandro Ghisla, Salim Al-Babili, Jean Cavarelli, Peter Beyer

**Affiliations:** 1 Faculty of Biology, Centre for Biological Signaling Studies, University of Freiburg, Freiburg, Germany; 2 Département de Biologie Structurale Intégrative, Institut de Génétique et Biologie Moléculaire et Cellulaire, UDS, CNRS, INSERM, Illkirch, France; 3 Department of Biology, University of Konstanz, Konstanz, Germany; University Paris Diderot-Paris 7, France

## Abstract

CRTI-type phytoene desaturases prevailing in bacteria and fungi can form lycopene directly from phytoene while plants employ two distinct desaturases and two *cis*-*tans* isomerases for the same purpose. This property renders *CRTI* a valuable gene to engineer provitamin A-formation to help combat vitamin A malnutrition, such as with Golden Rice. To understand the biochemical processes involved, recombinant CRTI was produced and obtained in homogeneous form that shows high enzymatic activity with the lipophilic substrate phytoene contained in phosphatidyl-choline (PC) liposome membranes. The first crystal structure of apo-CRTI reveals that CRTI belongs to the flavoprotein superfamily comprising protoporphyrinogen IX oxidoreductase and monoamine oxidase. CRTI is a membrane-peripheral oxidoreductase which utilizes FAD as the sole redox-active cofactor. Oxygen, replaceable by quinones in its absence, is needed as the terminal electron acceptor. FAD, besides its catalytic role also displays a structural function by enabling the formation of enzymatically active CRTI membrane associates. Under anaerobic conditions the enzyme can act as a carotene *cis*-*trans* isomerase. In *silico*-docking experiments yielded information on substrate binding sites, potential catalytic residues and is in favor of single half-site recognition of the symmetrical C_40_ hydrocarbon substrate.

## Introduction

Carotenoids are indispensible in photosynthetic energy metabolism both, in prokaryotes and eukaryotes, where they serve in light harvesting and photoprotection. In addition, β-carotene and oxygenated xanthophylls serve in plants as precursors in the formation of phytohormones, such as abscisic acid and the strigolactones [1], [2]. Some non-photosynthetic plant tissues containing chromoplasts, heterotrophic bacteria and fungi can also biosynthesize carotenoids *de novo*. Certain carotenoids possessing at least one unsubstituted β-ionone ring are essential in vertebrates, where they exert provitamin A-activity [3], besides additional health benefits that carotenoids can exert *per se* [4].

**Figure 1:**
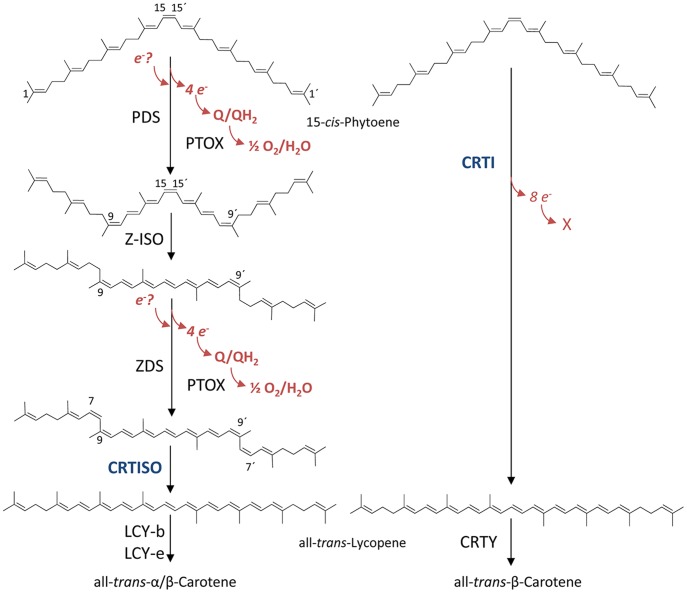
Phytoene desaturation – “complex” vs. “simple”. Left, the plant/cyanobacterial system consisting of the two desaturases, phytoene desaturase (PDS) and ζ-carotene desaturase (ZDS). The pathway involves specific poly-*cis*-intermediates and results in the formation of 7,9,9′7′-tetra-*cis*-lycopene ( =  prolycopene). *Cis*-*trans* isomerases act at the 9,15,9′-tri-*cis*-ζ-carotene (Z-ISO) and prolycopene (CRTISO) stage, the latter forming all-*trans*-lycopene, the substrate for lycopene cyclases. The electron acceptors identified so far for PDS (assumed here to be the same for the related ZDS) are plastoquinone and the plastoquinone:oxygen oxidoreductase PTOX. The necessity for an electron donating branch, resulting in redox chains into which PDS integrates has been suggested. Right, CRTI-mediated phytoene desaturation encompassing all four desaturation steps and one *cis*-*trans* isomerization step to form all-*trans-*lycopene. The desaturase CRTI and the isomerase CRTISO share sequential similarity.

Carotenoids are colored due to their polyene chromophore. These (mostly) eleven conjugated double bonds are formed from saturated precursors by carotene desaturases. As judged by sequence homology, carotene desaturation evolved at least twice (Figure 1). Cyanobacteria and plants employ a complex, multi-component pathway relying on two desaturases, namely phytoene desaturase (PDS) and ζ-carotene desaturase (ZDS), that form specific poly-*cis* configured carotene intermediates [5], [6] necessitating the participation of two *cis*-*trans* isomerases. These are ζ-carotene *cis-trans* isomerase (Z-ISO; [7], [8]) and carotene *cis*-*trans* isomerase (CRTISO; [9], [10]). The latter allows lycopene cyclase activity that acts as a non-permissive selectivity filter for lycopene *cis* isomers [11]. The plant-type carotene desaturation system is mechanistically linked to redox chains in which quinones [12], [13], the alternative oxidase PTOX [14] and molecular oxygen participate [5], [15]. This is contrasted by the phytoene desaturases of the CRTI-type prevailing in archaea, bacteria and fungi which are capable of catalyzing the entire desaturation sequence including one *cis*-to-*trans* isomerization reaction at the central double bond (Figure 1; for review, see [16]).

For the conversion of colorless 15-*cis-*phytoene into red-colored all-*trans-*lycopene CRTI thus takes over the function of at least four enzymes employed by cyanobacteria and plants. Based on this, CRTI N-terminally fused to a transit peptide allowing plastid-import has been expressed in crop plant tissues to successfully increase carotenoid/provitamin A levels such as in Golden Rice grains [17], [18], maize grains [19], tomato fruit [20] potato tubers [21] and – initially - in tobacco [22].

In contrast to such intense investigations carried out *in vivo*, studies dealing with CRTI enzymology are scarce and hindered by the fact that the substrate(s) and product are extremely hydrophobic C_40_ hydrocarbons located within the core of membrane systems [23]. We have explored a biphasic system containing the phytoene substrate embedded in phosphatidyl-choline liposomal membranes. This allowed very high conversion rates with purified CRTI from *Pantoea ananatis* (formerly *Erwinia uredovora*), overexpressed in *E. coli.* This has permitted us to gain insights into the CRTI-catalyzed reaction, to obtain structural information (PDB code: 4DGK; RCSB ID code: RCSB070301), to make statements on membrane topology and on putative substrate and cofactor binding sites.

## Results

### CRTI Purification and Enzymatic Activity

**Figure 2:**
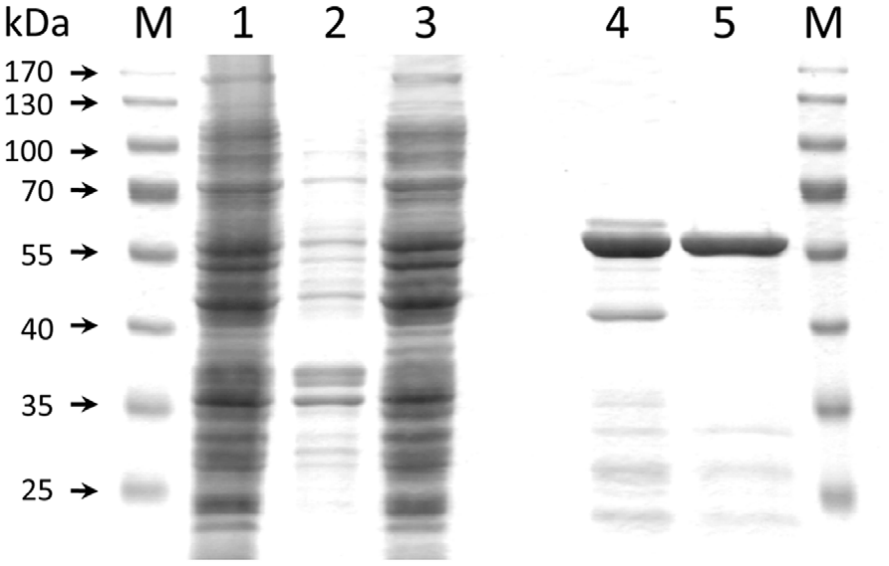
SDS-PAGE analysis of overexpressed CRTI protein: fractions and purification. The expected molecular mass of the overexpressed protein is 56 kDa. *Lane M*, molecular mass markers; *lane 1,* whole cell lysate after IPTG induction; *lane 2*, pellet after 12,000 × *g* centrifugation; *lane 3*, supernatant after 12,000 × *g* centrifugation; *lane 4,* fraction after IMAC purification; *lane 5*, fraction after GPC-purification.

**Figure 3:**
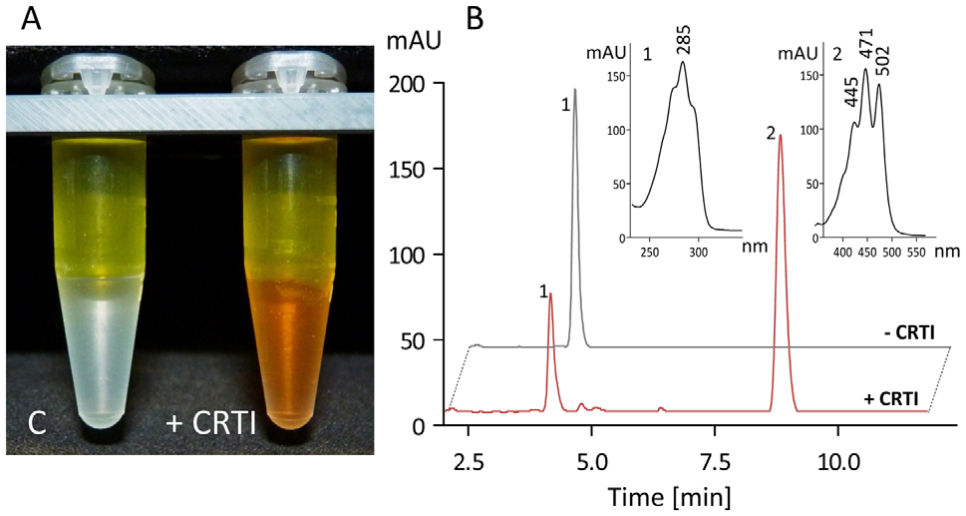
CRTI phytoene desaturation activity. A, standard incubation assay (see Experimental Procedures) extracted with CHCl_3_/MeOH 2∶1 (v/v) after an incubation time of 30 min. The yellow color in the aqueous epiphase is FAD. The organic phase contains the colorless phytoene substrate in the control (c, no CRTI added) or the red-colored lycopene (+15 µg CRTI). B, HPLC separation (system 1) of the organic phases shown in A (MaxPlot; peaks recorded at individual λ_max_). Insets show the corresponding UV/VIS spectra of phytoene (1) and all-*trans*-lycopene (2).

Overexpression in *E. coli* produced a substantial proportion of CRTI-His_6_ soluble protein allowing purification to near homogeneity by IMAC and subsequent gel permeation chromatography (GPC; Figure 2), where the protein eluted at the position expected for the monomeric form (56.04 kDa). No detergents were needed although CRTI must interact with the lipid bilayer. To test for enzymatic activity, 15-*cis*-phytoene-containing phosphatidyl-choline liposomes were made and supplemented with 15 µg of purified protein (see Experimental Procedures). This was enzymatically inactive unless supplemented with FAD, as revealed by the appearance of red color (Figure 3). The UV/VIS spectra of the organic extract showed formation of all-*trans-*lycopene. HPLC analysis revealed all-*trans*-lycopene formation at the expense of 15-*cis*-phytoene without accumulation of appreciable amounts of desaturation intermediates. Unlike the investigation of CRTI activity in *vivo* (see below), the formation of bisdehydrolycopene was not observed. Because of this uniform product formation, most of the kinetic data produced could be obtained through UV/VIS spectroscopy using HPLC as a confirmatory tool.

The reaction conditions of the standard incubation assays are the result of kinetic optimization. Purified CRTI showed a linear correlation of activity with protein concentration up to ca. 25 µg ml^-1^ (Figure S1A). A protein concentration of 21 µg ml^-1^ was therefore selected. At low pH CRTI is essentially inactive. With increasing pH the activity increases reflecting an apparent pK ≈ 6.3 and attains a plateau at pH >7 (Figure S1B).

**Figure 4:**
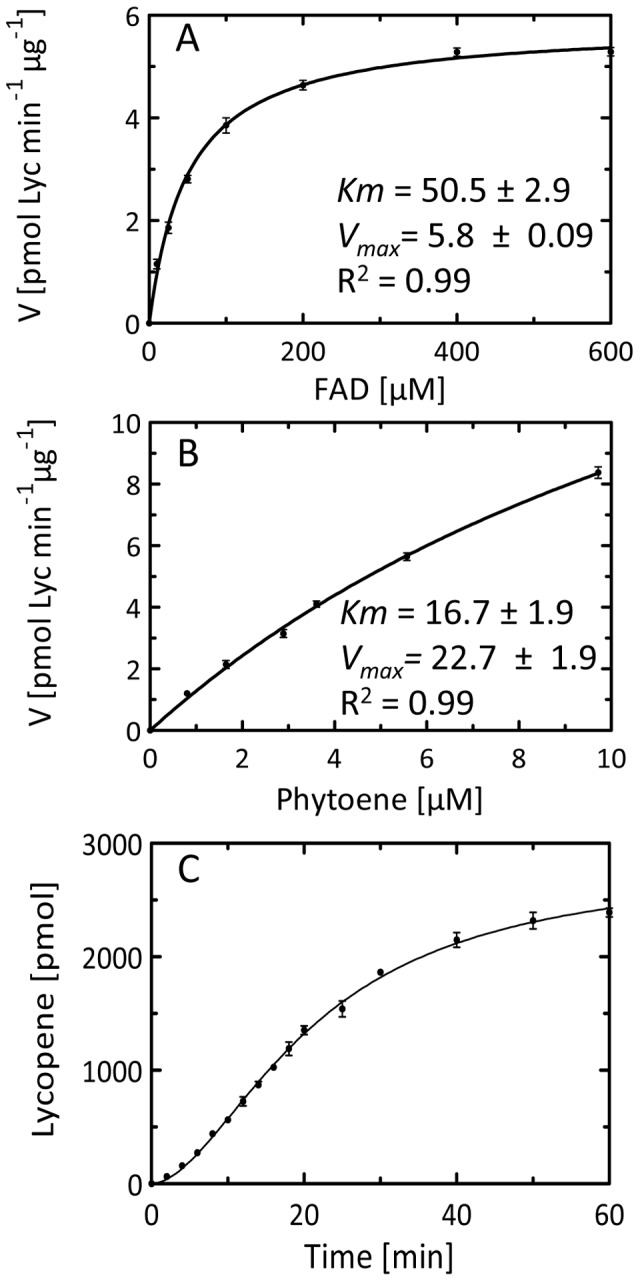
Characterization of CRTI phytoene desaturase activity. A, Dependence on the FAD concentration at a constant 7.5 µM phytoene concentration; B, variation of the phytoene concentration at a constant 150 µM FAD concentration. Phytoene liposomes were supplemented providing 0.8, 1.65, 2.89, 3.61, 5.57, 9.2 µM phytoene. Incubation time was 12 min at 37°C; C, Time course of lycopene formation in a standard incubation assay. Symbols represent the data from the mean of three replicate experiments (bars: ± SE). The curves through the data points in A and B are fits obtained with the Graphpad Prism software and using the equation Y =  V_max_^X/(Km+X)^.

FAD, FMN, NAD^+^ and NADP^+^ were tested as redox cofactors potentially capable of binding to the predicted Rossmann fold. Among these, only FAD was effective. Using a 12 min incubation time, and a 7 µM phytoene concentration an apparent *K_m_* of 50.5±2.9 µM was estimated for FAD (Figure 4A). A saturating FAD concentration of 150 µM was chosen for all further experimentation. To estimate the *K_m_* for phytoene a set of liposomes was made providing the apparent phytoene concentrations shown in Figure 4B (note that the “two dimensional” phytoene concentration in membranes will be considerably higher). It was not possible to achieve higher phytoene concentrations without interfering with the structural integrity of liposomes which then precipitated. A K*_m_* ≈ 16.7±1.9 µM was determined with a *V_max_* of ≈ 22±1.9 pmol min^-1^. A final concentration of 7 µM was chosen for routine studies because small liposomes formed most reliably and reproducibly maintaining their structure upon freezing. This parameter was important considering the accessible liposomal surface area and their predominantly unilamellar nature as determining factors for activity. Under these conditions, time courses for product formation were as shown in Figure 4C.

### Role of FAD and Nature of the Terminal Electron Acceptor

**Figure 5:**
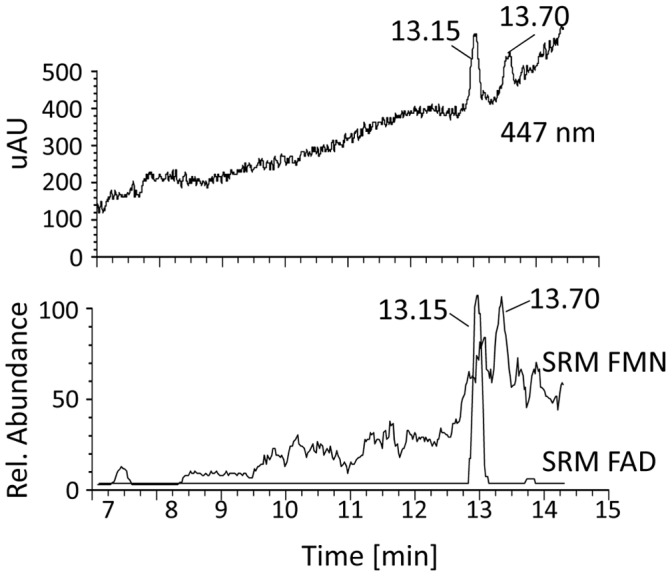
LC-MS-MS determination of CRTI-bound cofactors . Upper Trace, photometric response. Lower trace, Single Reaction Monitoring (SRM) was used to determine the presence of FAD M^+1^ = 786.2 MS^2^ daughter ions m/z  = 348.1, 439.2 and FMN M^+1^ = 457.1 MS^2^ daughter ions m/z  = 359.2, 439.1. The respective analyses for NAD(H) and NADP(H) yielded no signals. The separation was carried out using HPLC system 5.

Purified CRTI protein was essentially colorless and showed hardly detectable flavin fluorescence emission indicative of mainly apoprotein presence. We used LC-MS-MS Single Reaction Monitoring (SRM) as described previously [24] to investigate bound cofactors. Only FAD was detected at very low levels with some traces of FMN (Figure 5). Taking the data from above into account, we conclude that FAD is the sole cofactor effective in CRTI-mediated phytoene desaturation.

In order to obtain information on the occurrence of 2e^-^ or 1e^-^ transfer mechanisms, 5-deaza-FAD was used since it is active only in 2e^-^ redox chemistry [25]. We used this FAD-analog as reported previously with lycopene cyclase CRTY [24] and carotene *cis*-*trans* isomerase (CRTISO [11]). Both, under aerobic and anaerobic conditions (using duroquinone as electron acceptor, see below), CRTI was completely inactive with 5-deaza-FAD (150 µM).

FAD reoxidation occurs by oxygen because the enzyme proved to be fully inactive in dehydrogenation when equilibrated with nitrogen. Furthermore, since the assays were conducted in the presence of a 23-fold molar excess of FAD_ox_ compared to phytoene, it is concluded that the generated FAD_red_ is not released and exchanged with FAD_ox_ in the time scale of the reaction cycle. Thus, oxygen must play the role of a terminal electron acceptor.

**Figure 6:**
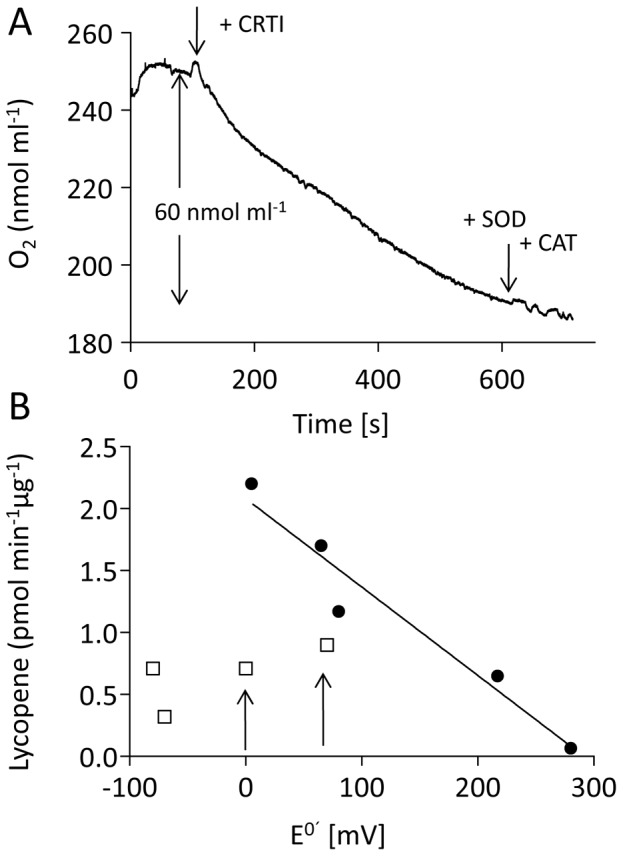
Electron transfer reactions catalyzed by CRTI. A, Potentiometric measurement of oxygen consumption during phytoene desaturation. B, Phytoene desaturation (lycopene formation) using quinones as electron acceptors. The assays were run under an N_2_ atmosphere for 30 minutes otherwise maintaining the standard conditions. The quinones used were menaquinone (−80 mV), phylloquinone (−70 mV), menadione (0 mV), duroquinone (+5 mV), Q10 (+65 mV), naphtoquinone (+70 mV) dichlophenolindophenol (+217 mV) and benzoquinone (+280 mV) all at a concentration of 240 µM. Open squares, naphtoquinones, filled symbols, benzoquinones.

Oxygen consumption was measured using an oxygen electrode in upscaled assays needed to attain the required sensitivity. Using 15 nmol phytoene and 250 µg (4.5 nmol) CRTI in 1 ml volume, oxygen consumption was ≈ 60 nmol within 10 min incubation time (Figure 6A). During this time a total of 8 nmol lycopene was formed generating 32 nmol of double bonds corresponding to the liberation of 64 nmol electrons (see Figure 1), i.e. to one e^-^ for each O_2_ consumed. This would suggest the formation of superoxide; however, the addition of superoxide dismutase and of catalase had no influence on the kinetics of oxygen consumption. We hypothesize that superoxide - if formed - might not be liberated; it could react with components of the liposomal membrane. CRTI thus behaves as a phytoene:oyxygen oxidoreductase when oxygen is present.

Quinones were investigated as alternative electron acceptors under anaerobic conditions. Naphtoquinones showed to be less effective than benzoquinones (Figure 6B) as evidenced by comparing menadione with duroquinone or Q10 with naphtoquinone with midpoint potentials of 0–5 mV and 65–70 mV, respectively (arrows). Optimal performance was obtained using quinones with a standard midpoint potential (E^0′^) of ca. 0–100 mV. Increase of the benzoquinones midpoint redox potentials correlated linearly with a decrease of the specific activity. This showed duroquinone (2.2 pmol lycopene µg^-1^ min^-1^) to be approximately as effective as oxygen (2.4 pmol lycopene µg^-1^ min^-1^).

### Flavin-binding and Membrane Association is one Concerted Process

**Figure 7:**
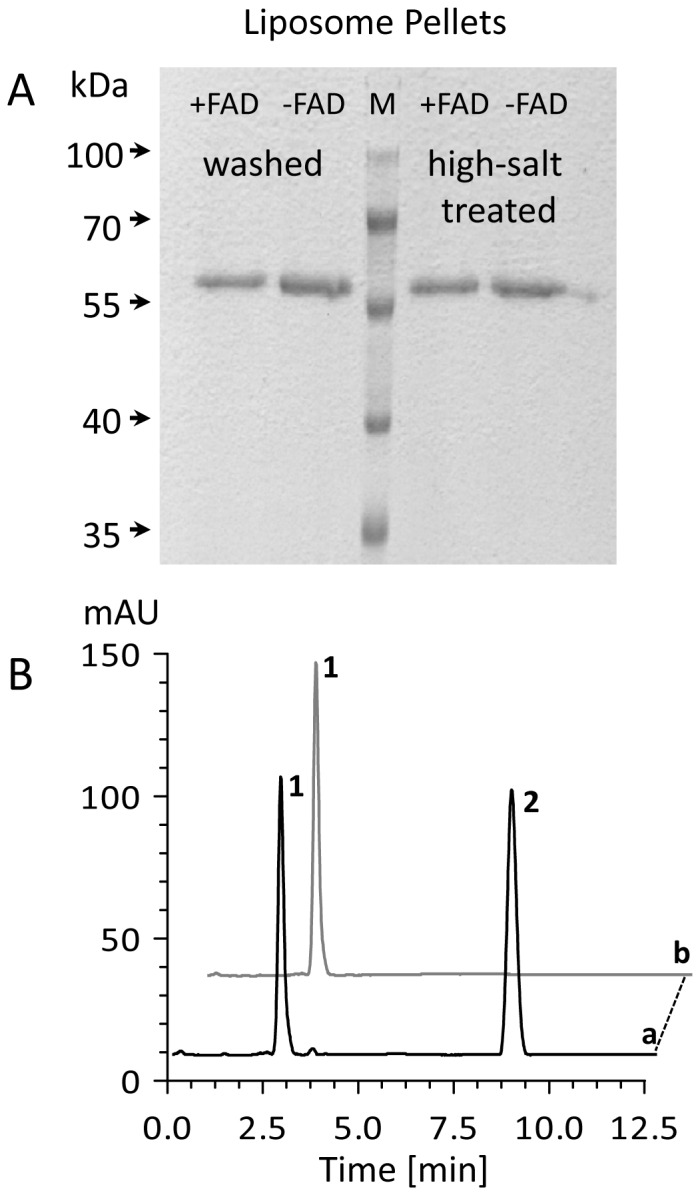
Effect of FAD on the formation of productive membrane associates. A, SDS-PAGE of liposome-bound CRTI. Membrane binding was carried out in the presence (+FAD) or absence of FAD (-FAD). CRTI bound to liposomes was analyzed after two washing steps with buffer II (left traces) or after a buffer II and additional high-salt washing step (right traces). B, HPLC analysis of phytoene desaturation catalyzed by membrane-bound CRTI. Trace a; lycopene (2) formation from phytoene (1) by membrane-associated CRTI formed in the presence of FAD but incubated without subsequently adding free FAD (free FAD removed by the washing steps). Trace b, same experiment using CRTI associates prepared in the absence of FAD; the incubation contained 150 µM added FAD. Incubation time was 1 h at 37°C. HPLC trace represents a MaxPlot (250–550 nm).

The apparent *K_m_* for FAD (≈ 50 µM) appears relatively high given the notion that it is practically not exchangeable during turnover (see above). In fact, holoenzyme formation appears to occur in a cooperative manner concomitant with its association to the membrane. This was shown by incubating 300 µg CRTI (5.3 nmol) at 37°C for 1h with 120 µl of substrate-free liposomes (930 nmol lipid) to exclude enzymatic activity during the preparative steps and in the presence or, separately, in absence of 500 µM FAD. Free FAD and unbound protein were removed and liposome-bound CRTI was recovered by centrifugation at 21,000×g at 4°C for 30 min. The two kinds of liposome pellets were washed first with 800 µl buffer II and subsequently with either buffer II or a 1 M KCl solution in buffer II. The resulting four pellets were subjected to SDS-PAGE analysis (Figure 7A). All samples showed a band for CRTI at 56 kDa.

For activity testing, protein-liposome pellets were resuspended in 100 µl buffer II and mixed with 100 µl of phytoene containing liposomes delivering 5 nmol phytoene and incubated for 1 h at 37°C. Samples in which CRTI had assembled with the membranes in the absence of FAD, were supplemented with FAD (240 µM) prior to activity testing. HPLC analysis of the extracted assays revealed that CRTI which had assembled to membranes in the presence of FAD was enzymatically active not requiring the addition of free FAD. In contrast, CRTI obtained by membrane association in the absence of FAD was inactive and could not be reactivated by subsequent addition of FAD (Figure 7B). This confirms that FAD release from active, membrane associated CRTI is comparatively slow, an exchange process during turn-over not being catalytically relevant. This also suggests that FAD plays a structural role enabling the formation of active CRTI membrane associates.

### Switching from Desaturase to *cis*-*trans* Isomerase Activity

**Figure 8:**
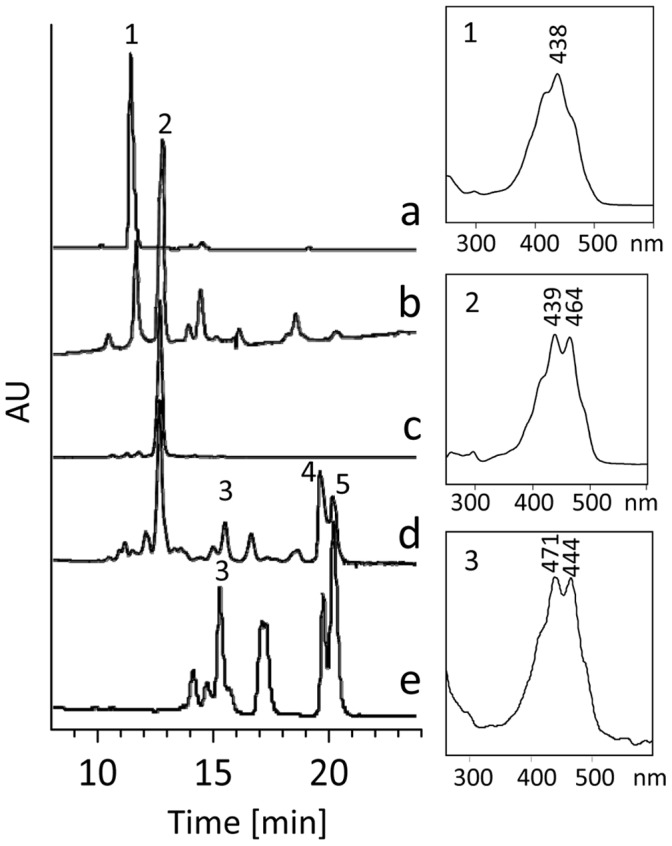
CRTI switch from desaturase to isomerase activity. Trace a, showing the elution profile (HPLC system 2) of prolycopene (1) extracted from the liposomes used. Trace b, profile of a 3 h incubation (37°C) of prolycopene in the presence of 30 µg CRTI and 100 µM FAD_red_ under anaerobic conditions. A tri-*cis*-lycopene species (2) forms predominantly. Trace c, tri-*cis*-lycopene was purified and incorporated into liposomes. Trace d, liposomes from c, analyzed after incubation with CRTISO and FAD_red_ [11]. This shows the formation of 7,9-di-*cis*-lycopene (3), all-*trans*-lycopene (4) and 5-*cis*-lycopene (5). Trace e, profile of the iodine-catalyzed isomer equilibrium obtained with the tri-*cis* species in organic solution. UV/VIS spectra of relevant *cis*-lycopene species are displayed on the right panels.

Phytoene desaturation mediated by CRTI also involves a *cis*-to-*trans* isomerization step of the central C15–C15′ double bond (Figure 1). Moreover, CRTI shares homology with CRTISO [9], the plant carotene isomerase for which an FAD_red_-dependent reaction mechanism was shown [11]. We therefore investigated whether the CRTI associated with reduced FAD formed transiently during phytoene desaturation can mediate phytoene *cis*-to-*trans* isomerization. This requires an anaerobic incubation regime of CRTI in the presence of FAD_red_ (see Experimental Procedures). No conversion of the membrane-bound 15-*cis-*phytoene substrate was observed (Figure S2). However, the CRTISO substrate prolycopene (7,9,9′,7′-tetra-*cis*-lycopene (for structure see Figure S7), when analogously incubated with CRTI, led to the formation of a novel tri-*cis-*lycopene species accompanied by smaller amounts of the half-side isomerized 7,9-di-*cis*-lycopene. The tri-*cis* structure was confirmed by converting the isolated tri-*cis* species into di-*cis* and all-*trans-*lycopene with the aid of the carotene *cis*-*trans* isomerase CRTISO and separately by treatment with catalytic amounts of iodine (Figure 8).

**Figure 9:**
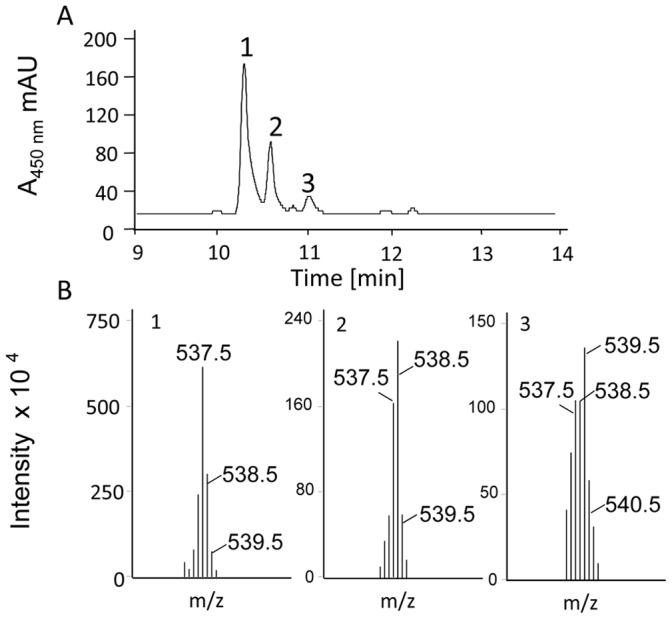
Prolycopene isomerization mediated by CRTI in the presence of ^2^H_2_O. A, HPLC analysis (HPLC system 4) showing the isomerization of tetra-*cis*-lycopene (prolycopene, peak 1) into tri-*cis*-lycopene (peak 2) and 7,9 di-*cis*-lycopene (peak 3) after a 3 h incubation (37°C) in the presence of 30 µg CRTI and 100 µM FAD_red_ under anaerobic conditions. B, mass spectra (numbering according to A) of remaining substrate and of products.

Whether isomerization reactions rely on acid-base catalysis can be assessed by carrying out the reaction in ^2^H_2_O and analyzing for label incorporation. Lycopene cyclase CRTY is an example [24]. Prolycopene isomerization reactions were carried out with CRTI under anaerobic conditions and in the presence of FAD_red_ (see Experimental Procedures) with all buffers and solutions made with ^2^H_2_O. LC-MS analysis revealed that the remaining tetra-*cis*-lycopene substrate was detected in the form of its M^+1^ quasi molecular ion at m/z  = 537.5 (Figure 9) accompanied by the expected ≈ 44% relative intensity for the A+1 (^13^C isotope) signal. This excludes for ^1^H/^2^H exchange during the incubation. In contrast, the “A+1″ signal was strongly increased in relative intensity in the tri-*cis* species due to deuterium incorporation, just like the “A+2″ signal at m/z  = 539.5 in the di-*cis* product (compare spectra 1, 2, 3). Intriguingly, complete disappearance of m/z 537.5 in the tri-*cis* species and of m/z 538.5 in the di-*cis* species was not observed. This reflects incomplete/partial ^1^H/^2^H exchange at the isomerization site and suggests acid-base catalysis as the catalytic principle.

### Overall CRTI Structure

Native, enzymatically active CRTI was crystallized in the space group P3_2_21 with one monomer in the asymmetric unit. The structure of apo-CRTI was solved at 3.0 Å by multiple anomalous dispersion using selenomethionine derivatives. The model was further refined against a 2.35 Å resolution native data set to an R-factor of 19.1% and an R-free of 22.9% (Table 1).

**Table 1:** X-ray data collection and refinement statistics.

	CRTI Native	CRTI SeMet			
Data collection					
		Peak	Inflection	Remote	
Beamline	Proxima 1	Proxima 1			
Wavelength (Å)	0.9770	0.9791	0.9794	0.9770	
Space group	*P*3_2_21	*P*3_2_21			
Unit cell parameters (Å)	a = b = 90.7, c = 130.0	a = b = 90.6, c = 130.2			
Resolution^(a)^ (Å)	30–2.35 (2.35–2.39)	30–3.0 (3.0–3.11)	30–3.0 (3.0–3.11)		30–3.0 (3.0–3.11)
*R*_merge_ (%)†	5.6 (35.3)	3.7 (13.6)	3.9 (15.5)		3.8 (13.4)
*I*/σ*(I)*	22.5 (4.4)	24.0 (7.6)	25.2 (6.6)		23.3 (6.7)
Completeness (%)	99.8 (99.8)	98.2 (99.5)	98.8 (100)		98.9 (100)
Multiplicity	6.5 (3.8)	3.1 (3.1)	3.2 (3.2)		2.9 (3.0)
No. of unique reflections^(b)^	26274	23578	23680		21508
				
Refinement					
*R*_work/_ *R*_free_ (%)	19.0/23.0				
R.m.s deviations					
Bond lengths (Å)	0.010				
Bond angles (°)	1.08				
Protein atoms	3199				
Ligand atoms	21				
Water molecules	67				
Wilson *B* (Å^2^)	68.8				
*B* factors (Å^2^)					
Protein	72.9				
Main chain	69.9				
Side chain	76.0				
Ligands	81.3				
Water	66.2				
Ramachandran plot (%)					
Most favoured	93.5				
Additionally allowed	6.5				
Generously allowed	0				
disallowed	0				

† *R*_merge_  =  Σ_h_Σ_i_|〈*I*_h_〉−*I*_h,i_|/Σ_h_Σ_i_ *I*_h,I_ where *I*_h,I_ is the i-th observed intensity of a measured reflection of Miller index h and |〈*I*_h_〉 is the average intensity of this unique reflection.

a Numbers in parentheses refer to the highest resolution shell. ^b^For the CRTI SeMet statistics (peak, inflection and remote), the Friedel pairs were not merged in all calculations.

**Figure 10:**
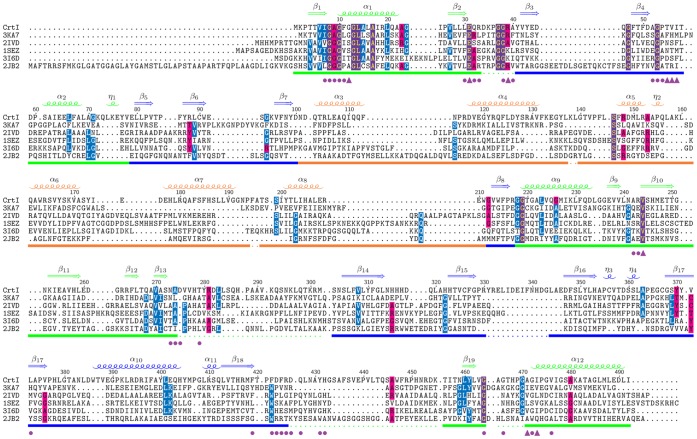
Structural alignment of CRTI with five FAD-binding Rossmann fold proteins (Pfam:CL0063) identified by a DALI search. The proteins are *Methanosarcina mazei* oxidoreductase (RMSD 4.6; 3 KA7; Seetharaman *et al*., unpublished), *Myxococcus xanthus* protoporphyrinogen oxidase (RMSD 4.6; 2 IVD; Corradi *et al*., 2006), *Nicotiana tabacum* mitochondrial protoporphyrinogen IX oxidase (RMSD 4.8; ISEZ; Koch *et al*., 2004), *Bacillus subtilis* protoporphyrinogen oxidase (RMSD 5.3, 3I6D, Qin *et al*., 2010) and *Rhodococcus opacus* L-amino acid oxidase (RMSD 4.8; 2JB2; Faust *et* *al*., 2007). The secondary structure elements of CRTI have been indicated above the alignment and the colored bar underneath the alignment indicates the domain organisation with the FAD-binding domain (green), the substrate-binding domain (blue), and the non-conserved ‘helical’ or ‘membrane-binding’ domain (orange). Disordered regions in the structure are represented by a dotted line and putative FAD binding residues are indicated by purple circles (hydrophobic interactions) and triangles (hydrophilic interactions). This figure was generated with TEXshade [60].

The structure of apo-CRTI is composed of 19 β-strands (forming 5 sheets), 12 alpha-helices, and three 3_10_-helices. Altogether these fold into three pseudo-domains consistent with the flavin containing amino oxidoreductase family (Pfam: PF01593) as revealed by structural search comparisons (see the topology diagram and structural alignment in Figure S3 and Figure 10, respectively). The first, the FAD binding domain is composed of a five-stranded, parallel sheet (sheet 1) sandwiched between a three-stranded anti-parallel sheet (sheet 5) and a five-helix bundle. The ligand binding domain is composed of a seven-stranded mixed topology sheet (sheet 4) with two alpha-helices packed onto the top surface and two, two-stranded anti-parallel sheets (sheets 2 and 3) and two 3_10_-helices packed onto one edge of the bottom surface of the sheet. The third domain packs against the rest of the bottom surface of sheet 4 and is composed of a six-helix bundle.

The final model showed several disordered regions, and of the 492 CRTI residues only 404 are visible in the electron density map. No density could be seen for residues 34–40, 138–139, 195–196, 275–302, 331–343, 426–455, 465–470. As described below, structural analyses revealed that most of the disordered regions (i.e. 34–40, 275–302, 426–455, 465–470) are involved in the predicted FAD binding site and therefore order-to-disorder transition should occur upon FAD binding and membrane binding (see above).

**Figure 11:**
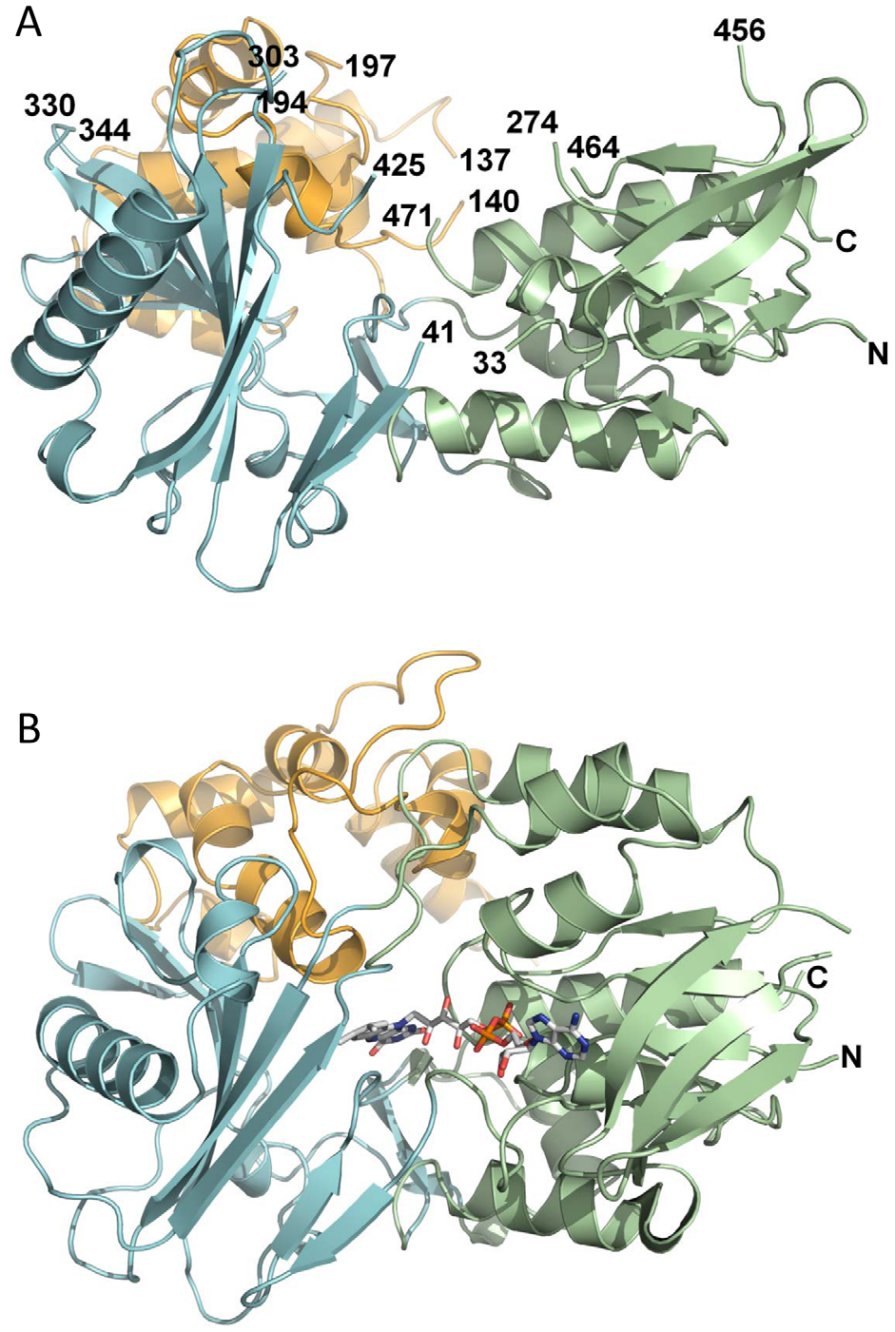
Structure of CRTI. The crystal structure of CRTI (A) is shown in comparison with protoporphyrinogen IX oxidoreductase from *Myxococcus xanthus* (B; Protein Data Bank 2IVD). Pseudodomains are colored in blue (substrate-binding), orange (non-conserved ‘helical’ or ‘membrane binding) and green (FAD-binding). Image was generated with PyMOL. The non-ordered regions in CRTI are indicated by the numbering of adjacent residues.

The structure of CRTI was submitted to the DALI server [26] for comparison with structures in the Protein Data Bank. All of the hits returned by DALI belong to the FAD/NAD(P)-binding Rossmann fold (Pfam: CL0063), although none showed more than 22% sequence identity with CRTI. Out of the top ten non-redundant hits, five bind FAD like CRTI (Table S1). These are *Methanosarcina mazei* oxidoreductase (RMSD 4.6; 3KA7; Seetharaman *et al*., unpublished), *Myxococcus xanthus* protoporphyrinogen oxidase (PPOX) (RMSD 4.6; 2IVD; [27], *Nicotiana tabacum* mitochondrial protoporphyrinogen IX oxidase (RMSD 4.8; 1SEZ; [28], *Bacillus subtilis* protoporphyrinogen oxidase (RMSD 5.3, 3I6D, [29] and *Rhodococcus opacus* L-amino acid oxidase (RMSD 4.8; 2JB2; [30]. A side-by-side comparison of CRTI with protoporphyrinogen oxidase from *Myxococcus xanthus* is shown in Figure 11. The whole protein, the FAD-binding, and the substrate-binding domains of the five FAD binding proteins were individually superimposed onto the structure of CRTI by secondary structure matching using LSQKAB from CCP4 [31] (Table S1). The non-conserved ‘helical’ or membrane binding domain was not superimposed as it shows considerable structural variability.

### Substrate and Cofactor Binding Analyses

Despite extensive efforts, attempts to produce crystals containing CRTI holoprotein for structure determination by soaking or co-crystallization were not successful. Addition of FAD prevented crystal formation and attempts to soak FAD into existing crystals led to their destruction. A reason for this may well lie in the cooperativeness of FAD-binding and membrane association shown above. Similarly, the substrate 15-*cis*-phytoene, soluble in water immiscible organic solvents, could not be incorporated. Therefore, in the absence of an experimental holo-structure, docking studies were performed. *In silico* ligand docking was unsuccessful with the entire FAD molecule and probably due to the structural differences between apo-and holoenzyme mentioned above. However, blinddock, followed by targetdock calculations (see Experimental Procedures) using the redox-active isoalloxazine resulted in a single cluster (200 out of 200 dockings) at −7.66 kcal binding energy (range of 0.02 kcal) showing the resulting structures as superimposed within a tunnel-like cavity as shown in Figure S4A. This position is analogous to that for isoalloxazine binding in PPOX (Figure 11B).

No meaningful docking results were obtained with 15-*cis* or all-*trans-*phytoene. In view of potential half-site recognition of the symmetrical C_40_ substrate, phytoene was successively truncated. Minimal energy clusters of binding interactions appeared only when the chain length was C_18_ to C_20_ (structures in Figure S7). Refined targetdock experiments resulted in a highly populated cluster (140 out of 200) at a binding energy of −7.21 kcal (range 1.85 kcal). Interestingly, the substrate molecules were within the same entry-tunnel as found for isoalloxazine (Figure S4B). Analogous *in silico* docking results were obtained with ligands of C_18_ to C_20_ chain length containing additional double bonds such as ζ-carotene or lycopene (structures in Figure S7).

**Figure 12:**
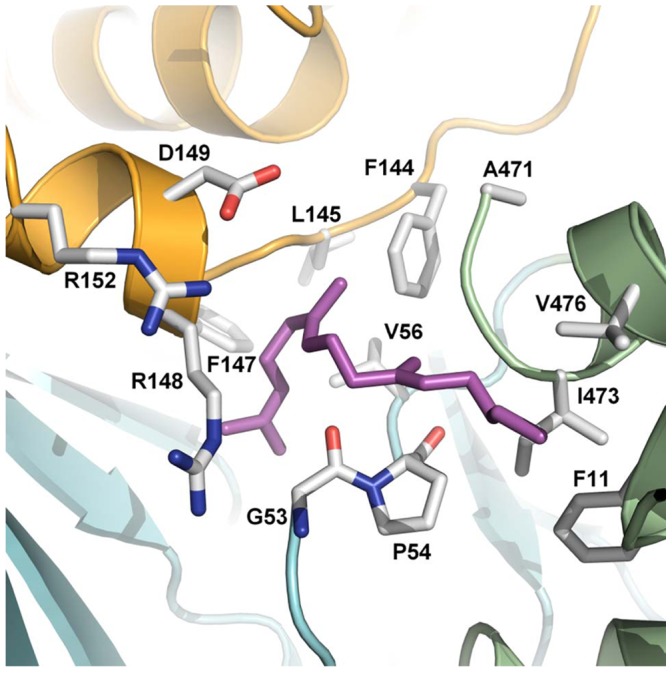
Substrate binding site and aligning amino acid residues. The substrate binding site shows the lowest energy conformations of *in silico*-docked C_18_ substrate. The isoalloxazine ring of FAD docks into the same site (Figure S4). The positions of substrate interacting hydrophobic residues are shown in grey (compare Table S2). See the Discussion for the likely role of the charged amino acids.

According to this model, hydrophobic residues capable of interacting with the hydrocarbon substrate are those shown in Figure 12 (see also Table S2). This also shows the presence of the charged residues D_149_, R_152_ and R_148_ at ≈ 5 Å from the substrate. These are supposedly activating functionalities (see Discussion).

A patch of conserved histidines (H_322_, H_323_ H_353_) line the end of the tunnel (Figure S5). This led us to suspect a coordination site for a metal cation as an additional redox center. We therefore mutated H_322_ and H_323_ to A and investigated the resulting CRTI_mut_ by complementing a phytoene-producing *E.coli* strain. Analyses of cells showed that desaturation products accumulated similarly with respect to quantity, but not quality. While CRTI produces significant amounts of bisdehydrolycopene *in vivo*, CRTI_mut_ was no longer able to introduce these additional two double bonds (Figure S6). Hence, these histidines are not catalytically active but can alter the active site geometry and hence, reaction specificity. Additionally, no Fe was detected by X-ray fluorescence scans and analysis of the electron density map did not reveal putative metal cofactor binding sites. Because their addition was also not required for activity, we conclude that there is no involvement of metal cofactors.

## Discussion

### CRTI- redox Reaction

The observation that highly purified phytoene desaturase CRTI-His_6_ from *Pantoea ananatis* very efficiently converts 15-*cis-*phytoene into all-*trans*-lycopene in the presence of FAD and in the absence of any other cofactors or metals speaks for FAD being the sole redox catalyst involved. This role of FAD is in agreement with previous results obtained with the same enzyme used in a combined assay in which a fungal protein extract was supplemented to introduce a [^14^C]phytoene generating system [32]. A CRTI-type phytoene desaturase from *Myxococcus xanthus* was also shown to contain FAD [33]. Since in CRTI-mediated desaturation *in vitro* redox equivalents are transferred from phytoene via FAD to oxygen the system behaves formally as an oxidase.

The absolute requirement for FAD is contrasted by the inability to obtain yellow recombinant protein. Despite numerous variations of the purification procedure including various detergents, only trace amounts of holoprotein were obtained. However, the membrane association experiments document a crucial structural role for FAD. It allows formation of enzymatically active, membrane associated CRTI, which is presumably accompanied by important structural changes. Conversely, while CRTI does bind to membranes in the absence of FAD, the resulting associate is inactive and incapable of subsequently binding FAD. Thus, while the membrane association process *per se* is FAD-independent, FAD presence during membrane-binding is crucial for holoenzyme formation. These findings parallel similar ones obtained with the plant-type phytoene desaturase PDS (see Figure 1), although its sequence similarity with CRTI is largely restricted to the N-terminal Rossmann fold region. PDS exists in an enzymatically inactive soluble HSP-70-bound form after plastid import. Activation was achieved upon FAD-binding concomitant with membrane-association. PDS activity was then independent of added FAD. Reconstitution in the absence of FAD resulted in an inactive membrane-bound apoprotein which could not be activated by subsequent addition of FAD [34]–[36].

The similarities between CRTI and PDS extend beyond this point. The activities of both depend *in vitro* on molecular oxygen [5], replaceable by quinones in its absence [12]. It is interesting to note that both enzymes utilize duroquinone optimally [15]. The activity of CRTI depends linearly on the redox potentials of the quinones used (Figure 6B). However, the decrease in rate with increasing potential is the opposite of the expected. This is compatible with the observed rate not reflecting the primary electron transfer, but a combination of kinetic steps of unknown nature. The analyses of Arabidopsis mutants impaired in carotene desaturation have confirmed quinones as the PDS electron acceptor *in vivo* [13]. Thus, the question arises whether the same holds true for CRTI when associated with other pathway enzymes. CRTI might then be functionally linked to the bacterial respiratory chain, as in the case of PDS that is thought to interact with redox chains [15], [37].

### *Cis-trans* Isomerase Activity

The sequence of carotene *cis*-*trans* isomerase (CRTISO) is related to that of CRTI [9], [10] and was shown to catalyze a non-redox reaction while requiring reduced FAD for activity [11]. Applying the incubation regime used for CRTISO to CRTI showed no *cis*-to-*trans* isomerization activity with 15-*cis*-phytoene. However, conversion of 7,9,9′,7′-tetra-*cis*-lycopene (prolycopene) into the asymmetrical tri- and the 7,9-di-*cis* species did occur. This is compatible with FAD playing a dual role. FAD_red_ can *cis-trans* isomerize (no net redox change), while FAD_ox_ acts as acceptor in a dehydrogenase (net redox) reaction. The structural differences determining the different properties of CRTI vs. CRTISO may be subtle and be related to the accessibility of oxygen.

CRTI containing FAD_red_ is able to *cis-trans* isomerize symmetrical tetra-*cis*-lycopene at one half side, corroborating the ideas developed below. This may be due to the *trans*-configuration of its central double bond. Central mono-*cis* carotenoids are thought to have both ends accessible from one side of the membrane [23]. It is conceivable that 15-*cis*-phytoene may allow the docking of two CRTI molecules to each “leg” of the symmetrical substrate (see below) in which case the *cis*-to-*trans* isomerization of the central *cis* double bond would be expected to be a later, if not the last step in the reaction sequence.

### Structure and Implications

The first crystal structure of CRTI described here adds strength to previous suggestions according to which a fingerprint motif located C-terminal of the conserved dinucleotide binding motif might allow placing carotene desaturases into a structural context with monoamine oxidases and protophorphyrinogen oxidases [33].

The superposition of the individual domains of five FAD-binding Rossmann fold proteins allowed for the identification of residues involved in FAD binding in CRTI (Figure 10). CRTI and mxPPOX share an overall 20% sequence identity and 35% sequence similarity. Of the twenty-nine residues implicated in FAD binding in mxPPOX (pdb code 2IVD), thirteen are invariant in CRTI with a further six residues being of similar type (45% identity, 66% similarity). Of the nine residues that make hydrophilic contacts with FAD only E39 (E31, CRTI) makes side-chain only contacts (with the ribose moiety) and is one of only six residues that are invariant in all six proteins (the others being G8, G10, G38, G53 and V244 in CrtI). Two others form main-chain and side-chain contacts, S20 and N441, but both are replaced by G12 and G466 in CRTI, allowing only the main-chain contacts to be preserved. Six other residues make main-chain only hydrophilic contacts. Of the twenty residues that make only hydrophobic contacts, eleven are invariant and a further four are similar. The superimposition of the individual domains is much better than the superimposition for the whole protein (Table S1) indicating that there may be domain movement upon FAD binding. In fact, the conformations of several of the key FAD binding residues (8–10, 53–54, 273–274, 464) vary considerably from those of the FAD bound structures. These residues probably change conformation upon FAD binding/membrane association along with most of the disordered regions which should fold over this site to form three helices (∼276–282, 291–298, and 442–457). The information from these superimpositions was used to manually edit the structural alignment produced by DALI which is shown in Figure 10.

The active site of mxPPOX sits in a tunnel that is formed between the ligand-binding domain and the membrane-binding domain. It runs all the way through the protein from the FAD binding site. In CRTI this tunnel is partially filled with aromatic residues, leaving a pocket composed of primarily hydrophobic residues (P54, T55, V56, F144, L145, S146, F147, R148, H323, Y351, H353, Y371). It is almost certain that this pocket would enlarge upon cofactor binding (residues 53–55 should be displaced to accommodate the isoalloxazine moiety) and it is possible that other structural rearrangements upon cofactor binding or membrane insertion could allow a channel to open.

CRTI has no membrane-spanning regions which argues against previous suggestions [38]. Contrary to expectations, the extremely hydrophobic carotenes are thus not being desaturated within, but at the surface of membranes. The mode of membrane-association may well be monotopic, as found with the apocarotenoid-15,15′-oxygenase [39] allowing substrate extraction from the lipid phase.

The location of FAD as suggested by the *in silico* studies is analogous to that found within the monoamine oxidase family. Similarly, the binding site of the carotene substrate in the vicinity of the isoalloxazine moiety appears related to the respective channels found e.g. in MAO and PPOX. Moreover, the vicinal histidines when mutated impact the number of double bonds inserted *in vivo*. A mutagenesis study conducted with a CRTI–type desaturase from *Rubriviax gelatinosus* pointed to L_208_ which also affected the number of double bonds inserted when mutated to Pro [38]. This corresponds to L_189_ in CRTI, which is located in some vicinity to the presumed substrate channel (Figure S5). Required catalytic residues can also be found within this site (see below). Altogether, this provides confidence for the authenticity of the identified site.

The results of i*n silico* docking are consistent with processing of half-sites of the symmetrical C_40_ aliphatic substrate. This appears plausible considering the length of the carotene chain which would not fit into the tunnel. There is precedence for such geometry in violaxanthin de-epoxidase which forms dimers, each monomer acting on a half-side of the symmetrical carotenoid [40], [41]. This implies that CRTI might be a dimer which, however, may only form on the surface of 15-*cis-*phytoene-containing membranes, when flavinylated and enzymatically active.

### Possible Mechanism of Dehydrogenation and Isomerization

Combining the structural data with those obtained from in *silico* docking allows formulating a working hypothesis for the mechanism of desaturation carried out by CRTI. Chemically, this reaction corresponds to the dehydrogenation of a -CH_2_-CH_2_- fragment that is flanked by a group capable of exerting activation (Scheme S1). This can be compared to the situation found in the αβ-dehydrogenation of acyl-CoAs, a reaction catalyzed by the intensively studied members of the family of the acyl-CoA dehydrogenases (ACADs) and acyl-CoA oxidases (ACOs) [42], [43]. This dehydrogenation is initiated by abstraction of the substrate α-H as a proton, the β-H then being transferred to the flavin as a hydride. Activation/acidification of the α-H is brought about by two strong H-bridges to the substrate thioester carbonyl (Scheme S1).

For CRTI, it is assumed that a functional group (∼B_2_ in Scheme S1) carries out a corresponding activation by (transiently) protonating the -C = C- double bond flanking the substrate α-H. In the ACAD/ACO family a carboxylate is the base abstracting the α-H. It is thus tempting to suggest a similar role for D_149_ (Figure 12) as the base B_1_. This would agree with the pH dependences of the activities (Figure S1B, [42]). In both enzyme groups the rates are low at low pH, where the carboxylate is likely to be protonated, and increase to a plateau with apparent pK’s near pH 7. Following abstraction of the α-H as a H^+^ the β-H would be transferred as a hydride onto the oxidized flavin. This could occur concertedly or via intermediates as with the ACADs and ACOs [42]. Possibly, the function of bases ∼B_1_ and ∼B_2_ could also be exerted by a single bidentate functionality. Candidate amino acids would be R_148_, D_149_ and R_152_ (Figure 11). Which of these are involved, acting singly or in combination, cannot be decided and must await corresponding mutagenesis of candidate amino acids singly and in combination.

The presence of a group that protonates a -C = C- function is also required for isomerization reactions in the presence of FAD_red_, which – in analogy to suggestions developed for CRTY and CRTISO [11], [24] - would serve as a stabilizer of an intermediate carbocation. There is an additional, mechanistically relevant similarity between CRTI and MCAD: Both enzymes reconstituted with 5-deaza-FAD are not active in dehydrogenation turn-over, although with MCAD transfer of a hydride to the cofactor occurs [44]. This would be consistent with a similar, basic mechanism of dehydrogenation for both enzyme classes.

## Materials and Methods

### Chemicals Used

^2^H_2_O was obtained from Euriso-top. All other fine chemicals were from Sigma-Aldrich. The purification and identification of prolycopene and of 5-deaza-FAD were carried out as described [11], [24].

### *CRTI* Cloning, Expression and Purification

*CRTI* was PCR amplified introducing a 5′ *Nde*I site and a 3′ His_6_-tag sequence as well as a *Hin*dIII site. This was cloned into pDGFHX, a pBR322 derivative, forming p*CRTI*-his.

Site directed double mutagenesis of H_322_ and H_323_ was performed by overlap extension PCR [45] using the primer pairs P_1_ CGGTAATTGGTGCAGGCTTCGGTGG, P1_mut_ GAAACAAACCGTGGCAGCCGCGAGCTGATC and P_2_ ACGGCCAGTGCCAAGCTTCAGTGAT, P2_mut_ : GATCAGCTCGCGGCTGCCACGGTTTGTTTC using p*CRTI*-his as a template. The resulting product was digested with *Bam*HI and *Hin*dIII and ligated into the respectively digested p*CRTI*-his vector yielding p*CRTI*mut.

*CRTI* was expressed as a C-terminal His_6_ fusion in *E. coli* JM109 (Promega). The cells were grown to an OD_600_ of 0.5 at 37°C, cooled down to 15°C, induced with 1 mM IPTG (isopropyl-1-thio-β-d-galactopyranoside) and incubated over night at 15°C. After harvest, 2–4 g cells were resuspended in 40 ml extraction buffer (50 mM Tris-HCl pH 8.0, 100 mM NaCl, 1 mM (tris(2-carboxyethyl)phosphine) (TCEP)) and subjected to 2 passes through a French Press at 20,000 psi. After centrifugation for 20 min at 12,000×g at 4°C, the soluble fraction was used for Immobilized Metal Affinity Chromatography (IMAC, TALON, Clontech). The purified protein was eluted with elution buffer (25 mM imidazole in extraction buffer). The protein was further purified by gel filtration using a HILOAD 16/60 SUPERDEX 200 PG column (GE Healthcare) with extraction buffer at a flow-rate of 1 ml min^−1^ using an Äkta Explorer FPLC (GE Healthcare). The protein was concentrated with Vivaspin2 (Heraeus, 10 kDa cut-off) and stored at −80°C. Protein was estimated using the Bradford reagent. The use of baffled flasks during bacterial growth was essential to produce enzymatically active CRTI.

Selenometionine-substituted CRTI was produced in *E. coli* [46] followed by protein purification as given above.

### *E. coli* System for Testing CRTI/CRTI_mut_ Activity *in vivo*

Phytoene-accumulating *E. coli* (TOP 10) cells were generated with the plasmid pPhytoene carrying a *Pantoea ananatis* phytoene synthesis cassette, harboring CRTE, ORF6 and CRTB. Phytoene-accumulating cells were then transformed with p*CRTI*-his or p*CRTI*mut and grown in 500 ml of LB medium containing kanamycin (50 µg ml^−1^) and ampicillin (100 µg ml^-1^) at 28°C to an OD_600_ of 0.5. protein expression was induced with 1 mM IPTG. Time courses were conducted with 50 ml aliquots and carotenoids extracted from the cell pellets by sonication in 2 ml acetone. The combined extracts were partitioned against 2 ml petroleum ether (PE) : diethyl ether (DE; 2∶1, v/v) and water. The dried carotenes were dissolved in 50 µl CHCl_3_; 5 µl aliquots were analyzed using HPLC system 1.

### Preparation of Phytoene Liposomes and Phytoene Desaturase Reactions

Phytoene was purified from phytoene-producing *E. coli* (see above) by extraction with acetone, followed by partitioning against PE : DE (2∶ 1, v/v) and water. The organic phase was dried, the residue dissolved in CHCl_3_ and subjected to a TLC purification step (silica gel 60 TLC plates, Merck) using PE:DE, 40∶10 (v/v) as the mobile phase. Phytoene was extracted from the solvent front with acetone and quantified spectrophotometrically in hexane (ε_285_ = 68,500 mol^-1 ^l^−1 ^cm^−1^).

Liposomes were prepared by dissolving 10 mg phosphatidyl-choline (Sigma-Aldrich) in 1 ml CHCl_3_. This was mixed with 100 nmol phytoene. The dried lipid-phytoene mixture was incubated with 2 ml buffer I (50 mM Tris-HCl pH 8.0, 100 mM NaCl) on ice for 30 min, then sonicated on ice for 5 min. Small unilamellar vesicles were formed by a French Press treatment at 20,000 psi [47].

For activity testing 15 µg purified CRTI was incubated with 100 µl phytoene liposomes (containing 5 nmol phytoene), 150 µM FAD and buffer II (50 mM phosphate buffer pH 8.0, 100 mM NaCl) in a total volume of 700 µl at 37°C for 30 min (standard assay). The reaction was started by adding the protein and stopped by adding 1 volume CHCl_3_ : MeOH (2∶ 1, v/v). After mixing, the sample was centrifuged at 20,000 x g for 10 min. The organic phase was used for reading UV/VIS spectra allowing lycopene quantification (ε_470nm_  = 171,255 mol^-1 ^l^-1 ^cm^-1^, in CHCl_3_). Alternatively, the organic phase was used for HPLC analysis.

### Desaturation Reactions in the Presence of Alternative Electron Acceptors

Long chain quinones (coenzyme Q10, decylplastoquinone, phylloquinone, menaquinone) were embedded into liposomes together with phytoene as described above. Quinone head-groups (duroquinone, menadione, p-benzoquinone, 1, 4-naphtoquinone) were added from ethanolic stocks. All quinones were added to a final concentration of 240 µM to the standard assay described above. Assays were conducted under an N_2_ atmosphere using N_2_-equilibrated solutions.

### *Cis-trans* Isomerization of Prolycopene (7,9,9′,7′tetra-*cis*-lycopene)

The CRTI-mediated isomerization of prolycopene was done in an N_2_ atmosphere using N_2_-equilibrated solutions. The standard CRTI-prolycopene assay (final volume 400 µl) consisted of 326 µl buffer II which was supplemented with 40 µl of the prolycopene-containing liposome to result in a final carotene concentration of 5 µM and with 30 µg of CRTI. FAD (100 µM, final concentration) was reduced by supplementing the assay with 4 µl of a freshly prepared 0.1M dithionite solution. Incubation took under an N_2_-atmosphere in the dark at 37°C for 3 h. The reaction was stopped by mixing with one volume of CHCl_3_/MeOH 2∶ 1 (v/v).

Non-enzymatic isomerization of lycopene isomers was carried out by dissolving *cis*-carotenes in n-hexane containing 0.0075% iodine. The solution was exposed to ambient light for 3 min.

For deuteration experiments, all components were prepared as described but in ^2^H_2_O. The reactions were carried out under standard anaerobic conditions and analyzed by LC-MS, as described earlier [24].

### Extraction and HPLC Analysis

The organic phases from enzymatic assays were dried and dissolved in CHCl_3_ for HPLC analysis using a Waters Alliance 2695 or an UFLC Shimadzu Prominence system, both equipped with a photodiode array detector (PDA).

HPLC system 1 was used for the separation of carotene substrates and products employing a 3 µm C_30_ reversed phase column (YMC-Europe) with the solvent system A: MeOH/tert-butylmethylether (TBME)/water 5∶1:1 (v/v/v) and B: MeOH/TBME 1∶1 (v/v). The gradient started at 43% A, followed by a linear gradient to 0% A within 5 min at a flow-rate of 0.7 ml min^-1^. An isocratic segment, run for 10 min at 0% A, completed the run.

HPLC system 2 was used for the identification of lycopene *cis*-isomers according to [48], [11]. The system consisted of a direct phase column (Nucleosil 300-5, Macherey & Nagel) with water-free hexane/N-ethyldiisopropylamine 2000∶ 1 (v/v) as the mobile phase used at a flow-rate of 1.5 ml min^-1^.

HPLC system 3 was used to baseline separate all-*trans*-phytoene from its 15-*cis* isomer employing a 3 µm C_30_ reversed phase column (YMC-Europe) with the solvent A: MeOH/TBME/water 5∶1:1 (v/v/v) and B: MeOH/TBME 1∶3 (v/v) at an isocratic flow-rate of 1.4 ml min^−1^ at 50% A.

HPLC system 4 was used for LC-MS (Thermo-Fisher LTQ) analysis of carotenes, as previously described [24]. Carotenes were APCI-ionized using N_2_ as reagent gas and analyzed in the positive ion mode.

HPLC system 5 was used for the analysis of NADP(H), NAD(H), FAD, FMN, and of flavin analogs by LC-MS. The compounds were separated and identified by MS^2^-dependent Single Reaction Monitoring (SRM) [24].

### Crystallization, X-ray Data Collection and Structure Solution

CRTI was concentrated to 9.5 mg ml^−1^ and crystallization conditions were screened using commercially available kits (Hampton Research and Qiagen suites). Experiments were carried out using the sitting-drop vapor-diffusion method in 96-well plates at 290 K. 200 nl protein solution and 200 nl precipitant solution were equilibrated against 50 µl reservoir solution. After optimization, diffraction quality crystals (200×150×150 µm) were obtained from hanging-drop vapor diffusion experiments in 24-well VDX plates (Hampton Research), where 2 µl of protein was mixed with 2 µl reservoir solution and equilibrated against 0.5 ml of reservoir.

Crystals were obtained with 8% PEG 8K, 0.1 M NaCl, 0.1 M Na/K phosphate pH 6.2 and crystals of the selenomethionine substituted protein were obtained with 6% PEG 8K, 0.1 M NaCl, 0.1 M Na/K phosphate pH 6.2. Crystals were transferred to cryoprotection buffer (6–8% PEG 8K, 0.1 M NaCl, 0.1 M Na/K phosphate pH 6.2, 20% ethylene glycol) and flash frozen in liquid nitrogen. Data were collected at 100K at the Proxima 1 beamline of Synchrotron Soleil, France, using a Quantum 315r CCD detector (ADSC, USA). Few crystals yield diffraction data better than 2.5 Å resolution using synchrotron radiation. Native data were collected to 2.35 Å and data were collected to 3.0 Å from selenomethionine substituted crystal at the absorption peak (0.9791 Å), inflection point (0.9794 Å), and a remote wavelength (0.9770 Å). All data were indexed, integrated and scaled using HKL2000 [49]. Data collection and refinement statistics are summarized in Table 1. Initial phase determination was performed by the MAD method using the program SHARP [50] followed by solvent flattening in SOLOMON [51]. Examining the electron density maps allowed the choice of space group as P3_2_21. The program BUCCANEER [52] was used to automatically locate structural fragments and a large part of the remaining model could be constructed in COOT [53]. 1299 reflections (4.9%) were randomly selected for *R*_free_ calculation. Refinement was carried out in REFMAC5 [54] initially against the Se-Met peak data, with the resolution being increased in stages to 2.35 Å for refinement against the native data. The final stages of refinement were carried out in BUSTER [55] and PHENIX [56]. The structure was refined using three TLS domains. After each round of refinement the structure was validated with MOLPROBITY [57] and the final structure validated with PROCHECK [58]. All other crystallographic calculations were carried out with the CCP4 suite [31].

### In *silico* Docking

FAD, isoalloxazine and carotene substrates were docked into the crystal structure using the AUTODOCK 4.0 software [59]. The docking parameters were calculated using the AUTODOCK standard procedures. Potential grid maps were calculated with a cubic box and a distance between grid points of 0.375 Å in targetdock and 0.5 Å in blinddock calculations. “Blinddock” simulations refer to calculations in which the grid box encompassed the entire protein. In “targetdock” simulations the grid box was narrowed down stepwise to a protein domain previously identified in blinddock experiments. The Lamarckian Genetic Algorithm with 25 million energy evaluations was applied as a search method. 200 docking experiments were carried out in each simulation. The docking results were evaluated by cluster analysis at a root mean square deviation cutoff of 2 Å. Structures were visualized using the PyMol Molecular Grapics System, version 1.3, Schrödinger, LLC.

## Supporting Information

Figure S1 **Dependence of the CRTI activity on the protein concentration (A) and pH (B).** The assays were carried out under standard incubation conditions as given in the Experimental Procedures section. The line through the data points in (B) is a fit based on the pH equation, it was forced to approach 0 at pH <5 and was generated with the KaleidaGraph. The curve reflects a pK  = 6.3±0.1.
(TIF)

Figure S2 **CRTI cannot isomerize 15-** ***cis-*** **phytoene into the all-** ***trans*** **form.** Enzymatic assays were carried out in the presence of 30 µg CRTI and 100 µM FAD_red_ under anaerobic conditions at 37°C with predominantly 15-*cis-*phytoene, accompanied by small amounts of the all-*trans* isomer as the substrate, incorporated into liposomes. The use of HPLC system 3 allowed baseline separation. Unlike with 7,9,9′,7′-tetra-*cis*-lycopene (prolycopene), no isomerization activity was observed.
(TIF)

Figure S3 **Topology diagram of CRTI.** The FAD-binding domain is colored green, the substrate-binding domain is colored blue, and the ‘helical’ or ‘membrane-binding’ domain is colored yellow. The FAD-binding domain is composed of a five-stranded, parallel sheet (sheet 1: β1 4–7; β2 27–30; β9 237–239; β13 270–272; β19 460–462) sandwiched between a three-stranded anti-parallel sheet (sheet 5: β10 244–250; β11 253–259, β12 264–266) and a five-helix bundle (α1 10–22; α2 61–68; η1 74–76; α9 220-233; α12 473-491). The substrate-binding domain is composed of a seven-stranded mixed topology sheet (sheet 4: β6 87-91; β7 96-99; β14 305-313; β15 323-329; β16 346-353; β17 368-376; β18 413-419) with two alpha-helices packed onto the top surface (α10 386-404; α11 409-412) and two, two-stranded anti-parallel sheets (sheet 2: β3 42-45; β4 48-51 and sheet 3: β5; 80–83; β8 213-216) and two 3_10_-helices (η2 355-357; η3 360-362) packed onto one edge of the bottom surface of the sheet. The third domain packs against the rest of the under surface of the sheet and is composed of a six-helix bundle (α3 103-113; α4 117-132; α5 147-152; α6 161-171; α7 177–191; α8 201–208). Putative FAD binding regions are highlighted in red and disordered regions are represented by a dashed line.
(TIF)

Figure S4 ***In silico*** **docking places the isoalloxazine ring and the carotene substrates into the same tunnel-like site.** A, all 200 simulations showed the isoalloxazine ring superimposed into the site given; the lowest energy conformation is shown. B, shows two representatives of the lowest energy cluster obtained for the C_18_ phytoene fragment. Very similar results were obtained using analogously truncated desaturation intermediates containing additional double bonds i.e. a C_18_ ζ-carotene and C_18_ lycopene fragment.
(TIF)

Figure S5 **Substrate tunnel and mutated amino acid residues.** Substrate tunnel cut open showing two lowest energy conformations of *silico*-docked C_18_ substrates. The positions of histidines at the bottom of the tunnel and of L_189_ are shown (see text for details).
(TIF)

Figure S6 **Production of bisdehydrolycopene is abolished in a H_322, 323_ double mutant**. *E*. *coli* culture aliquots were harvested at time points after IPTG induction and analyzed by HPLC. In *vivo* (but not in *vitro*) the wild-type (wt) CRTI is capable of introducing two additional double bonds to form bisdehydrolycopene (for structures see Figure S7). This capability is abolished in the mutated (mut) version. Blue, bisdehydrolycopene; red, lycopene; orange, 13-*cis*-lycopene, yellow, 15-*cis*-lycopene; green, ζ-carotene.
(TIF)

Figure S7 **Carotene structures and truncated carotenes used for in silico docking procedures.** 1, 15-*cis*-phytoene; 2, all-*trans*-phytoene; 3, all-*trans*-lycopene; 4, all-*trans*-bisdehydrolycopene; 5, 7,9,9′,7′-tetra-*cis*-lycopene (prolycopene); 6, C18-phytoene; 7, C18-ζ-carotene; 8, C18-lycopene.
(TIF)

Table S1 **Top ten non-redundant hits from a DALI search with the structure of CRTI.** 3D superimposition (RMSD calculation on Cα atoms after 3D superimposition using LSQKAB from CCP4):^ 1^Overall superimposition, ^2^superimposition using only the FAD-binding domains, ^3^superimposition using only the substrate-binding domains.
(DOCX)

Table S2 **Presumed hydrophobic substrate interacting amino acid residues.** ^(1)^Identity refers to 200 accessions grouping into eleven subgroups including *Bacteria* and *Archaea*. Multiple alignements and phylogenetic trees were obtained using the PipeAlign server at http://bips.u-strasbg.fr/PipeAlign/. ^(2)^The distance between residues and the docked carotene substrate was determined in PyMol. ^(3)^With the exception of *Archaea*.
(DOCX)

Scheme S1 **Proposed mechanism of dehydrogenation by CRTI and comparison to that of acyl-CoA dehydrogenases and oxidases.** Center panel: Active site arrangement in MCAD as discussed in [42]. The 2 H-bonds shown to interact with the CoA substrate carbonyl group are connected to the polypeptide back-bone and to the FAD C2’-OH. This forms an oxyanion hole-like set-up; the active site glutamate initiates dehydrogenation by abstraction of the αC-H as H^+^. Top panel: Analogous set-up for dehydrogenation by CRTI: ∼B_1_ is a base, possibly D_149_ that serves in abstracting the shown C-H as H^+^. ∼B_2_ is a positively charged group, possibly either R_148_ or R_152_ that serves in the polarization of the C = C double bond thereby activating/acidifying the neighboring C-H functionality. Bottom panel: Isomerization is acid-base catalyzed; FAD is retained in its reduced form serving as a stabilizer of the carbocation formed. See text for further details.
(TIF)
