# Supplementary figures and images for: On the Structure and Function of the Phytoene Desaturase CRTI from Pantoea ananatis, a Membrane-Peripheral and FAD-Dependent Oxidase/Isomerase
Source: PLoS One. 2012 Jun 22;7(6):e39550. doi: 10.1371/journal.pone.0039550 (PMC3382138; doi:10.1371/journal.pone.0039550)

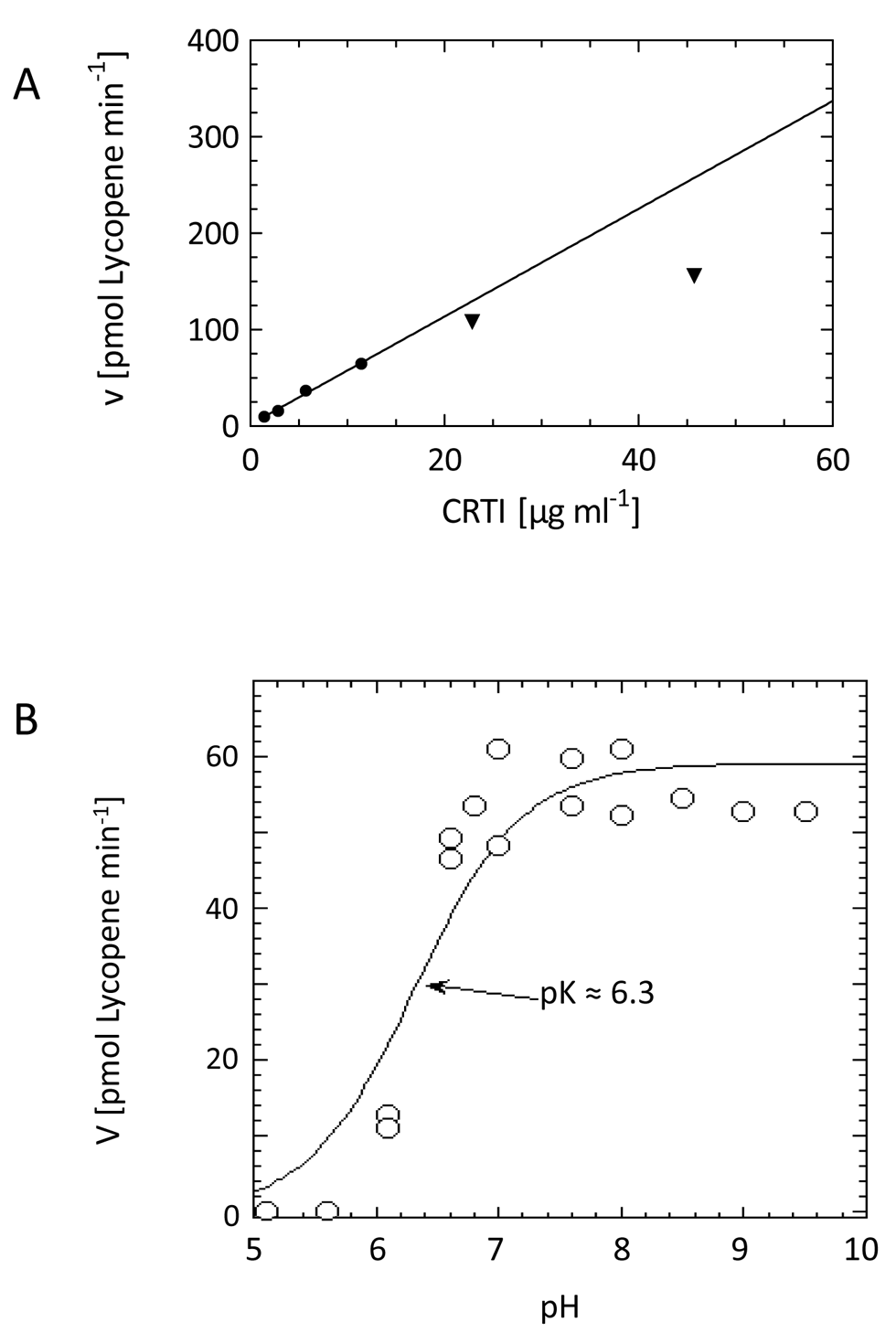

Supplement: Figure S1 — Dependence of the CRTI activity on the protein concentration (A) and pH (B). The assays were carried out under standard incubation conditions as given in the Experimental Procedures section. The line through the data points in (B) is a fit based on the pH equation, it was forced to approach 0 at pH <5 and was generated with the KaleidaGraph. The curve reflects a pK = 6.3±0.1. (TIF) [file pone.0039550.s001.tif]

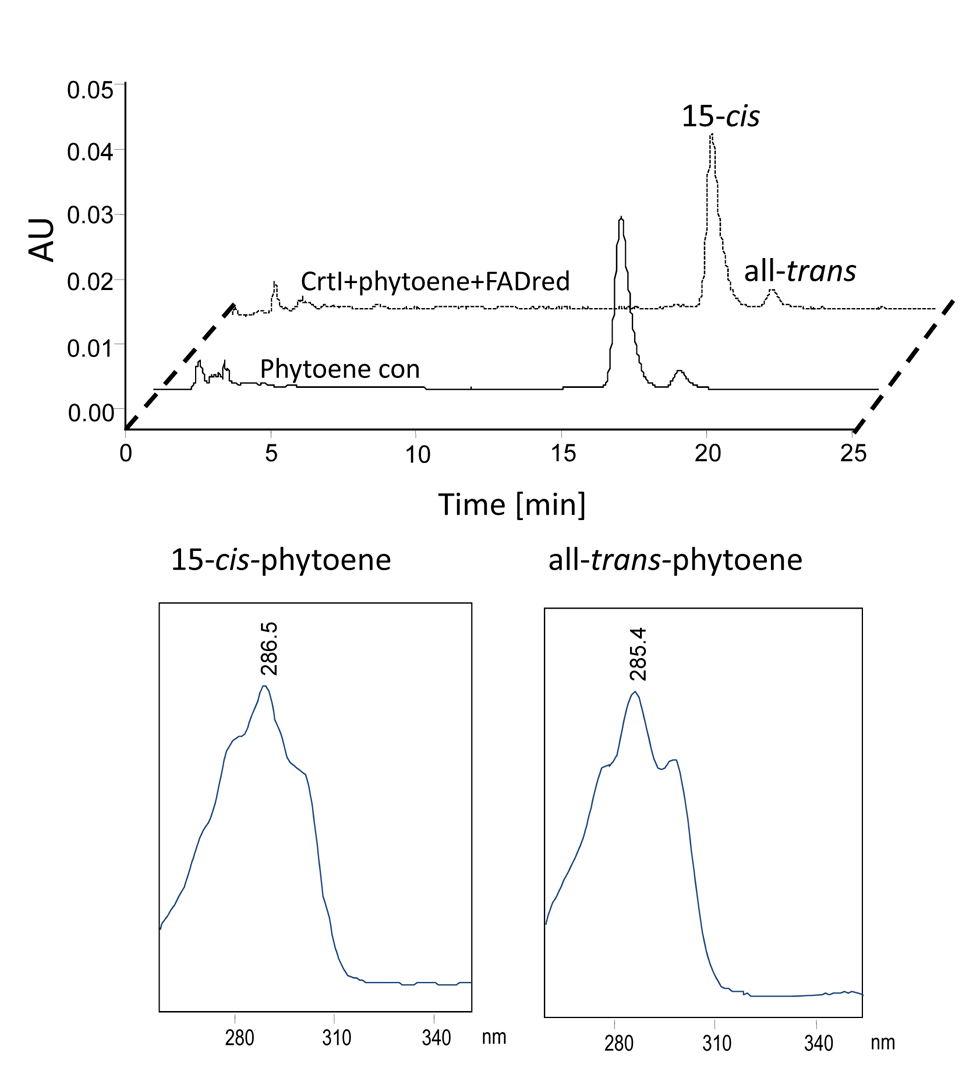

Supplement: Figure S2 — CRTI cannot isomerize 15- cis- phytoene into the all- trans form. Enzymatic assays were carried out in the presence of 30 µg CRTI and 100 µM FADred under anaerobic conditions at 37°C with predominantly 15-cis-phytoene, accompanied by small amounts of the all-trans isomer as the substrate, incorporated into liposomes. The use of HPLC system 3 allowed baseline separation. Unlike with 7,9,9′,7′-tetra-cis-lycopene (prolycopene), no isomerization activity was observed. (TIF) [file pone.0039550.s002.tif]

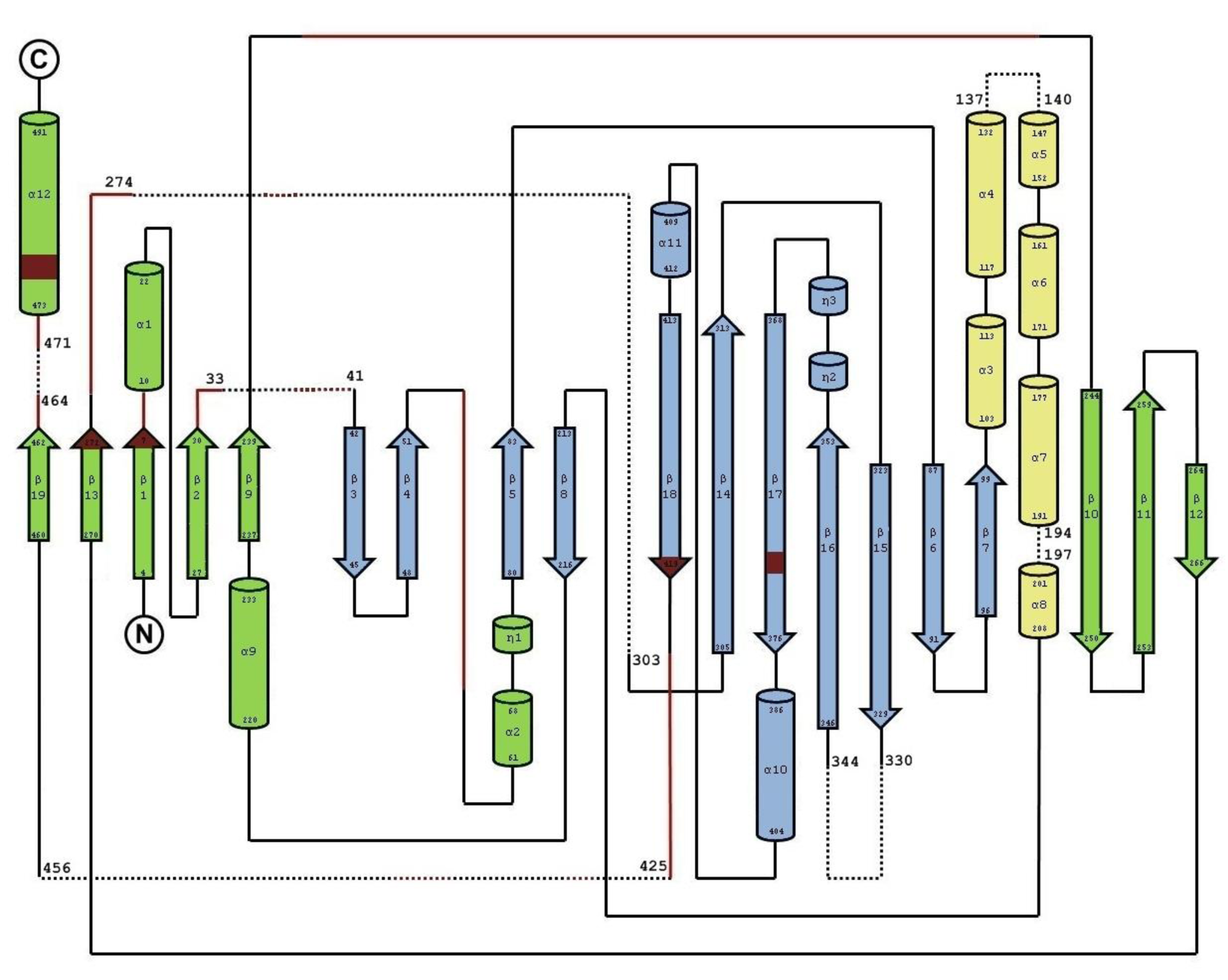

Supplement: Figure S3 — Topology diagram of CRTI. The FAD-binding domain is colored green, the substrate-binding domain is colored blue, and the ‘helical’ or ‘membrane-binding’ domain is colored yellow. The FAD-binding domain is composed of a five-stranded, parallel sheet (sheet 1: β1 4–7; β2 27–30; β9 237–239; β13 270–272; β19 460–462) sandwiched between a three-stranded anti-parallel sheet (sheet 5: β10 244–250; β11 253–259, β12 264–266) and a five-helix bundle (α1 10–22; α2 61–68; η1 74–76; α9 220-233; α12 473-491). The substrate-binding domain is composed of a seven-stranded mixed topology sheet (sheet 4: β6 87-91; β7 96-99; β14 305-313; β15 323-329; β16 346-353; β17 368-376; β18 413-419) with two alpha-helices packed onto the top surface (α10 386-404; α11 409-412) and two, two-stranded anti-parallel sheets (sheet 2: β3 42-45; β4 48-51 and sheet 3: β5; 80–83; β8 213-216) and two 310-helices (η2 355-357; η3 360-362) packed onto one edge of the bottom surface of the sheet. The third domain packs against the rest of the under surface of the sheet and is composed of a six-helix bundle (α3 103-113; α4 117-132; α5 147-152; α6 161-171; α7 177–191; α8 201–208). Putative FAD binding regions are highlighted in red and disordered regions are represented by a dashed line. (TIF) [file pone.0039550.s003.tif]

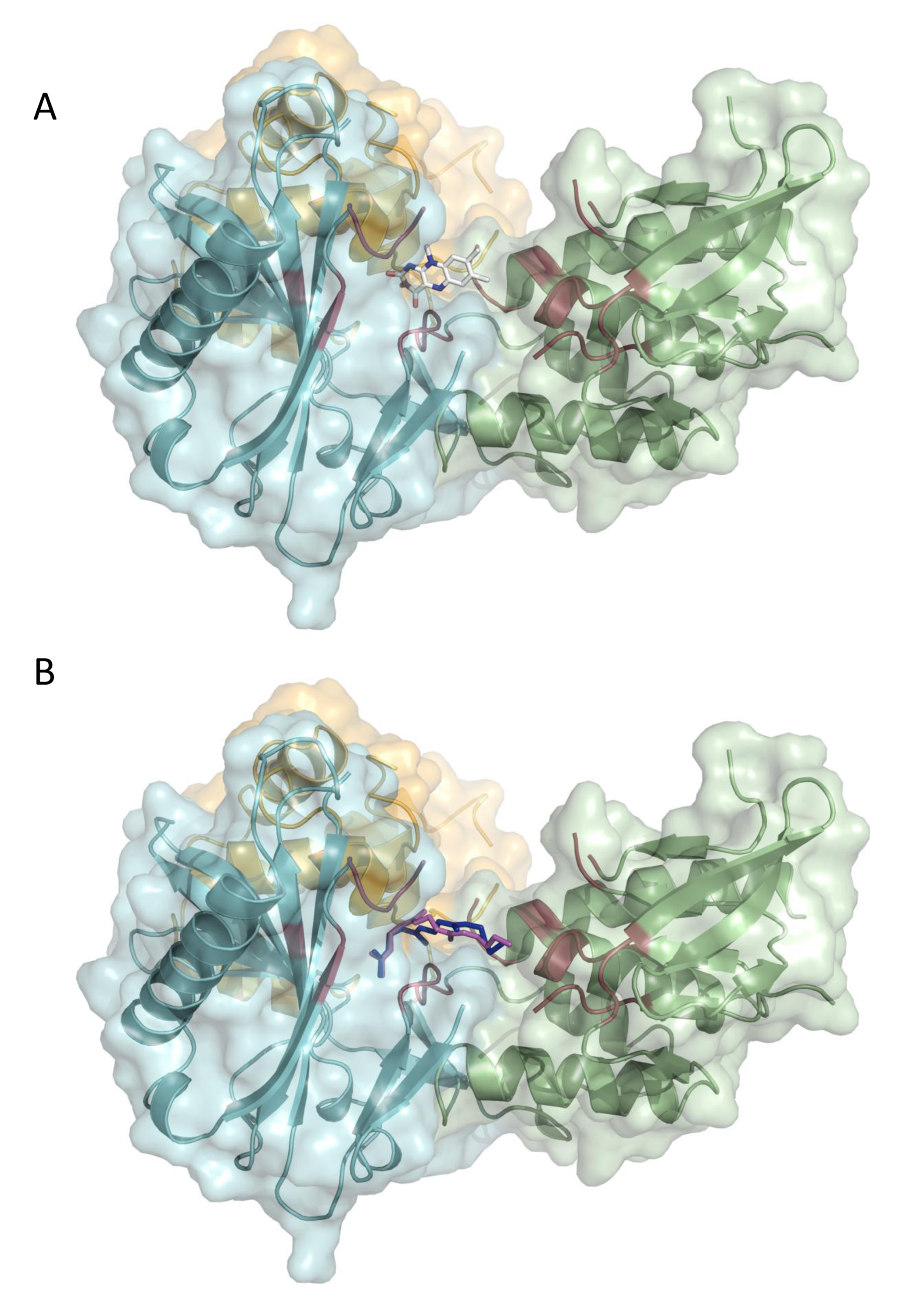

Supplement: Figure S4 — In silico docking places the isoalloxazine ring and the carotene substrates into the same tunnel-like site. A, all 200 simulations showed the isoalloxazine ring superimposed into the site given; the lowest energy conformation is shown. B, shows two representatives of the lowest energy cluster obtained for the C18 phytoene fragment. Very similar results were obtained using analogously truncated desaturation intermediates containing additional double bonds i.e. a C18 ζ-carotene and C18 lycopene fragment. (TIF) [file pone.0039550.s004.tif]

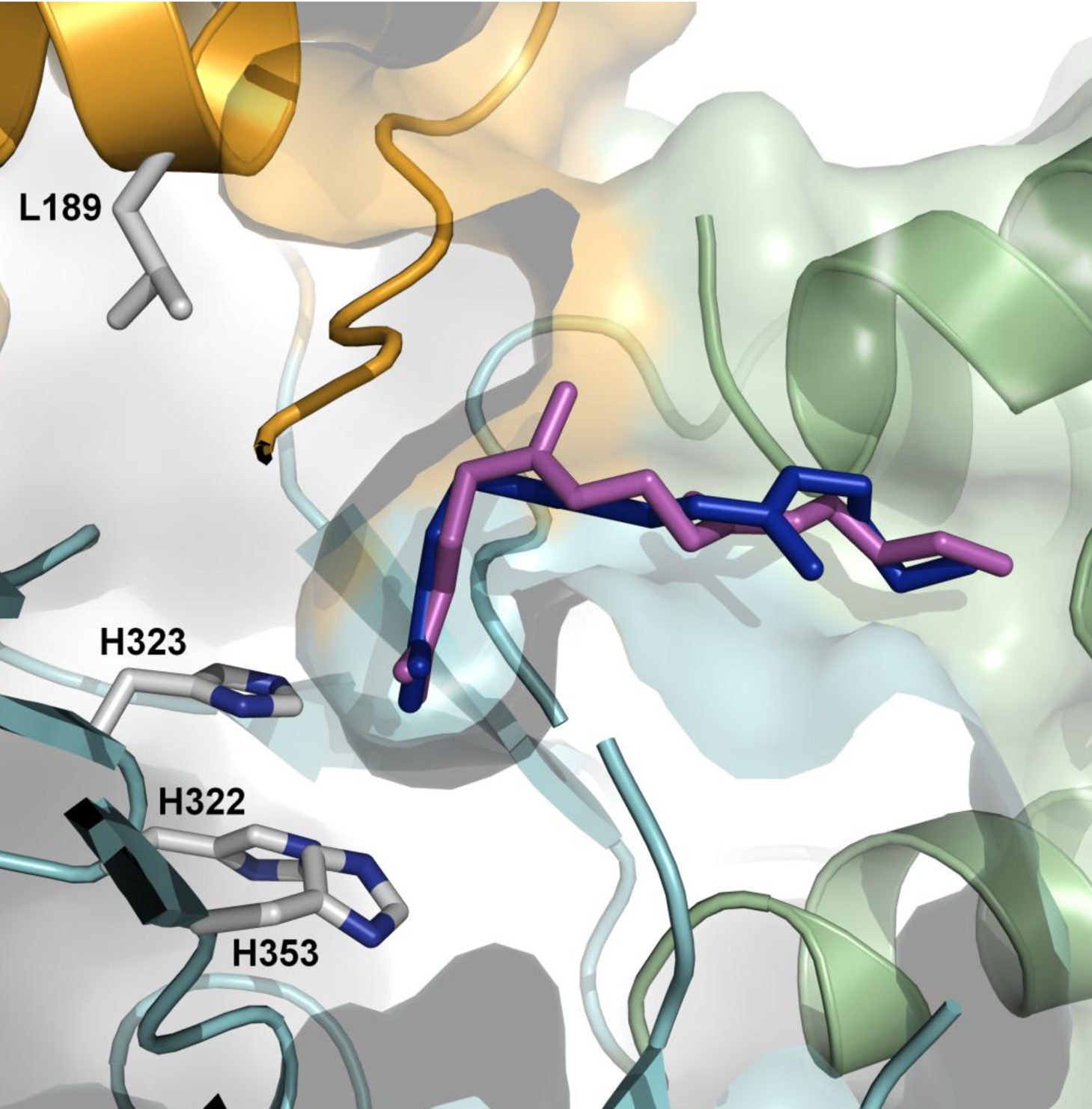

Supplement: Figure S5 — Substrate tunnel and mutated amino acid residues. Substrate tunnel cut open showing two lowest energy conformations of silico-docked C18 substrates. The positions of histidines at the bottom of the tunnel and of L189 are shown (see text for details). (TIF) [file pone.0039550.s005.tif]

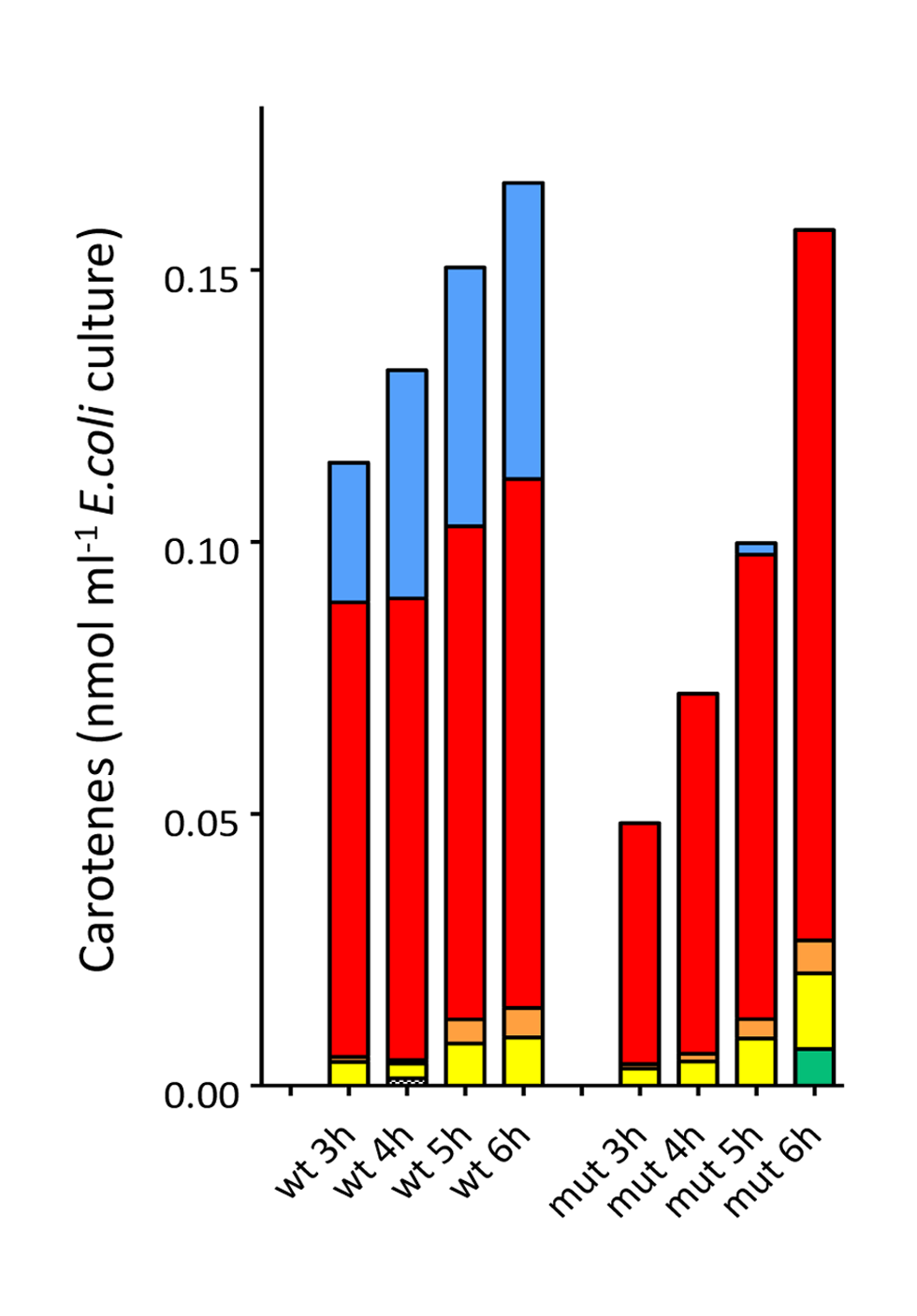

Supplement: Figure S6 — Production of bisdehydrolycopene is abolished in a H322, 323 double mutant. E. coli culture aliquots were harvested at time points after IPTG induction and analyzed by HPLC. In vivo (but not in vitro) the wild-type (wt) CRTI is capable of introducing two additional double bonds to form bisdehydrolycopene (for structures see Figure S7). This capability is abolished in the mutated (mut) version. Blue, bisdehydrolycopene; red, lycopene; orange, 13-cis-lycopene, yellow, 15-cis-lycopene; green, ζ-carotene. (TIF) [file pone.0039550.s006.tif]

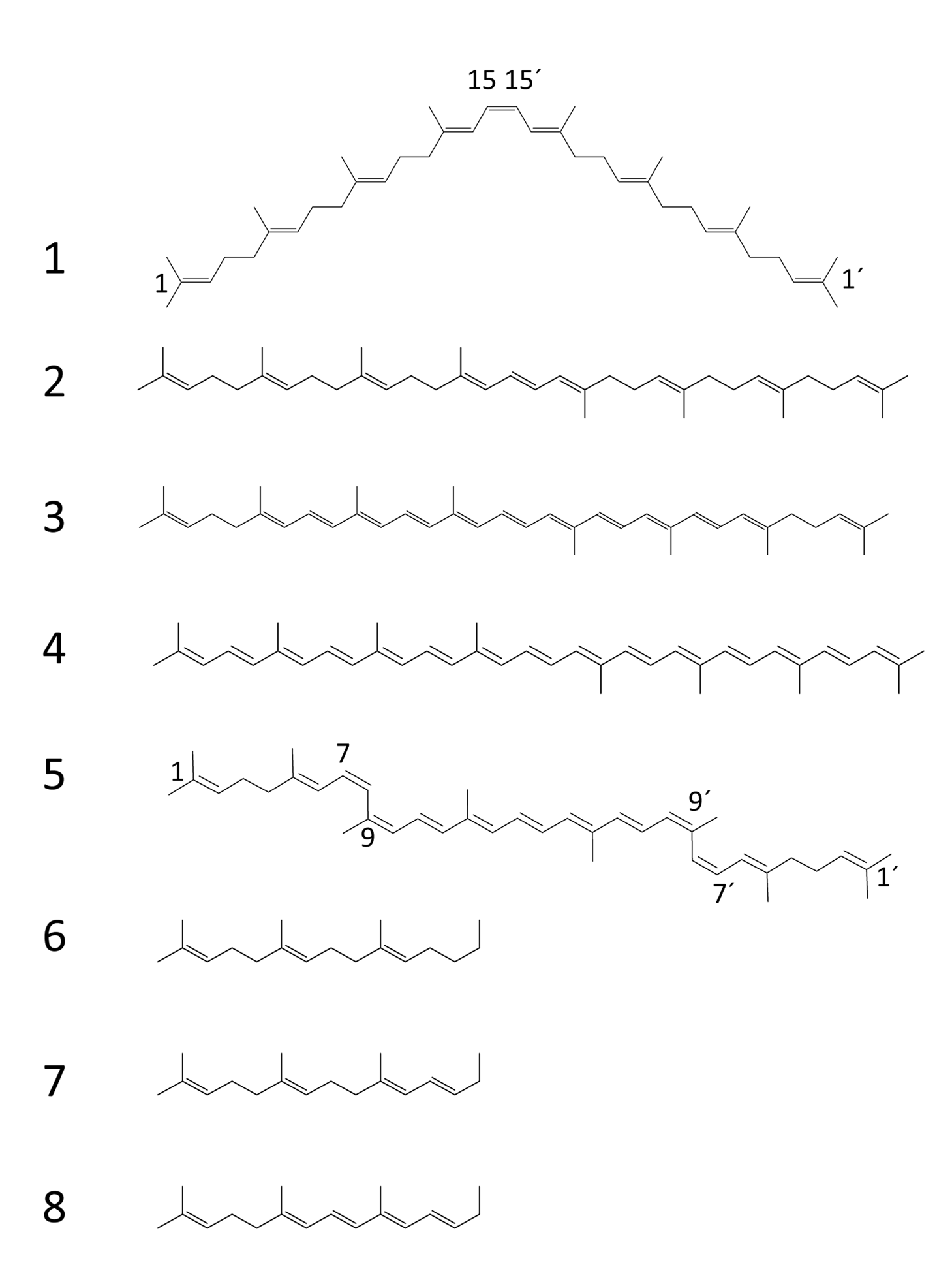

Supplement: Figure S7 — Carotene structures and truncated carotenes used for in silico docking procedures. 1, 15-cis-phytoene; 2, all-trans-phytoene; 3, all-trans-lycopene; 4, all-trans-bisdehydrolycopene; 5, 7,9,9′,7′-tetra-cis-lycopene (prolycopene); 6, C18-phytoene; 7, C18-ζ-carotene; 8, C18-lycopene. (TIF) [file pone.0039550.s007.tif]

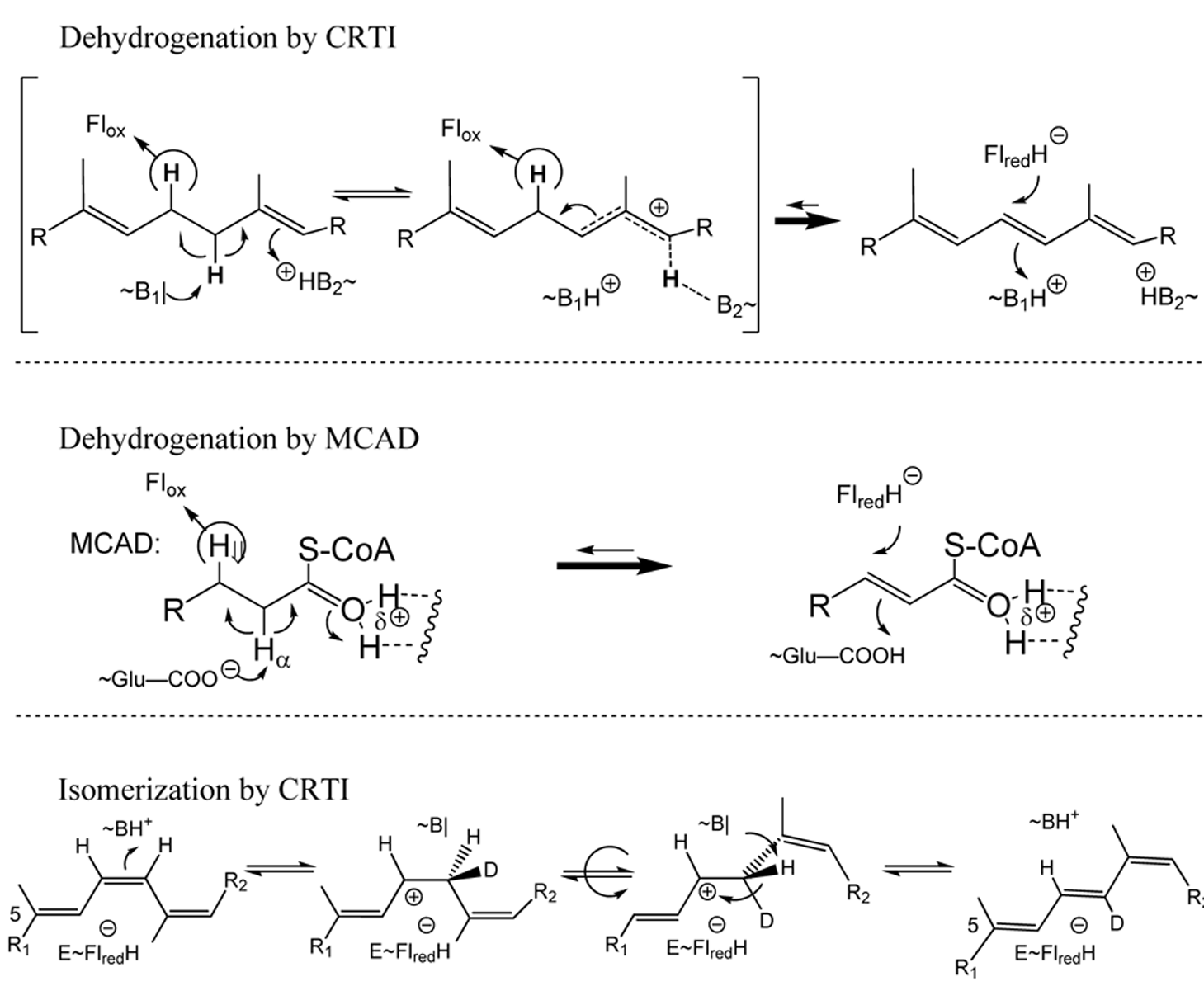

Supplement: Scheme S1 — Proposed mechanism of dehydrogenation by CRTI and comparison to that of acyl-CoA dehydrogenases and oxidases. Center panel: Active site arrangement in MCAD as discussed in [42]. The 2 H-bonds shown to interact with the CoA substrate carbonyl group are connected to the polypeptide back-bone and to the FAD C2’-OH. This forms an oxyanion hole-like set-up; the active site glutamate initiates dehydrogenation by abstraction of the αC-H as H+. Top panel: Analogous set-up for dehydrogenation by CRTI: ∼B1 is a base, possibly D149 that serves in abstracting the shown C-H as H+. ∼B2 is a positively charged group, possibly either R148 or R152 that serves in the polarization of the C = C double bond thereby activating/acidifying the neighboring C-H functionality. Bottom panel: Isomerization is acid-base catalyzed; FAD is retained in its reduced form serving as a stabilizer of the carbocation formed. See text for further details. (TIF) [file pone.0039550.s010.tif]
