# Supplementary material for: On the Structure and Function of the Phytoene Desaturase CRTI from Pantoea ananatis, a Membrane-Peripheral and FAD-Dependent Oxidase/Isomerase
Source: PLoS One. 2012 Jun 22;7(6):e39550. doi: 10.1371/journal.pone.0039550 (PMC3382138; doi:10.1371/journal.pone.0039550)
Supplement: Table S1 — Top ten non-redundant hits from a DALI search with the structure of CRTI. 3D superimposition (RMSD calculation on Cα atoms after 3D superimposition using LSQKAB from CCP4): 1Overall superimposition, 2superimposition using only the FAD-binding domains, 3superimposition using only the substrate-binding domains. (DOCX) [file pone.0039550.s008.docx]

**Table S1**  **Top ten non-redundant hits from a DALI search with the structure of CRTI**

| **Hit** | **PDB ID** | **Z score** | **^1^RMSD (all)** | **Identity (%)** | **Resolution (Å)** | **Cofactor** | **^2^RMSD (FAD-binding domain)** | **^3^RMSD (Substrate-binding domain)** |
| --- | --- | --- | --- | --- | --- | --- | --- | --- |
| 1 | 3KA7 | 21.4 | 4.6 | 18 | 1.8 | FAD | 2.018 | 2.508 |
| 2 | 3NRN | 20.8 | 4.2 | 19 | 2.1 | AMP |  |  |
| 4 | 1UKV | 20.4 | 4 | 14 | 1.5 | GDP |  |  |
| 7 | 1GND | 20.2 | 4.1 | 14 | 1.81 |  |  |  |
| 13 | 2IVD | 19.6 | 4.6 | 17 | 2.3 | FAD | 2.042 | 2.777 |
| 18 | 3P1W | 19.4 | 4.2 | 12 | 1.85 |  |  |  |
| 19 | 1VG0 | 19.4 | 3.5 | 14 | 2.2 | GDP |  |  |
| 21 | 1SEZ | 18.7 | 4.8 | 17 | 2.9 | FAD | 1.943 | 2.691 |
| 20 | 3I6D | 18.8 | 5.3 | 16 | 2.9 | FAD | 1.905 | 2.571 |
| 22 | 2JB2 | 18.7 | 4.8 | 15 | 1.45 | FAD | 1.918 | 2.886 |

3D superimposition (RMSD calculation on Cα atoms after 3D superimposition using LSQKAB from CCP4):^. 1^Overall superimposition, ^2^superimposition done using only the FAD-binding domains, ^3^superimposition done using only the substrate-binding domains
