# Supplementary material for: On the Structure and Function of the Phytoene Desaturase CRTI from Pantoea ananatis, a Membrane-Peripheral and FAD-Dependent Oxidase/Isomerase
Source: PLoS One. 2012 Jun 22;7(6):e39550. doi: 10.1371/journal.pone.0039550 (PMC3382138; doi:10.1371/journal.pone.0039550)
Supplement: Table S2 — Presumed hydrophobic substrate interacting amino acid residues. (1)Identity refers to 200 accessions grouping into eleven subgroups including Bacteria and Archaea. Multiple alignements and phylogenetic trees were obtained using the PipeAlign server at http://bips.u-strasbg.fr/PipeAlign/. (2)The distance between residues and the docked carotene substrate was determined in PyMol. (3)With the exception of Archaea. (DOCX) [file pone.0039550.s009.docx]

**Table S2 Presumed hydrophobic substrate interacting amino acid residues.**

| Residue | Identity^(1)^ [%] | Conservative  Exchange | Distance [Å]^(2)^ |
| --- | --- | --- | --- |
| F_11_ | 69 | + | 3.2 |
| P_54_ | 100 |  | 3.3 |
| V_56_ | 57 | + | 3.3 |
| F_144_ | 84 | or Y | 3.3 |
| F_147_ | 0 | 100 % | 3.9 |
| A_471_ | 89 | - | 5.2 |
| I_473_ | 0 | 100 %^(3)^ | 3.6 |
| V_476_ | 100 |  | 3.9 |

^(1)^Identity refers to 200 accessions grouping into eleven subgroups including *Bacteria* and *Archaea*. Multiple alignements and phylogenetic trees were obtained using the PipeAlign server at http://bips.u-strasbg.fr/PipeAlign/. ^(2)^The distance between residues and the docked carotene substrate was determined in PyMol. ^(3)^With the exception of *Archaea*.
